# Machine learning-assisted DFT-prediction of pristine and endohedral doped (O and Se) Ge_12_C_12_ and Si_12_C_12_ nanostructures as anode materials for lithium-ion batteries

**DOI:** 10.1038/s41598-024-77150-x

**Published:** 2024-10-31

**Authors:** ThankGod C. Egemonye, Tomsmith O. Unimuke

**Affiliations:** Department of Pure and Applied Chemistry, University of Calabar, PMB 1115, Calabar, Nigeria

**Keywords:** Nanocage, Anode, Lithium-ion battery, DFT, Machine learning, Chemistry, Energy science and technology, Nanoscience and technology, Materials science

## Abstract

Nanostructured materials have gained significant attention as anode material in rechargeable lithium-ion batteries due to their large surface-to-volume ratio and efficient lithium-ion intercalation. Herein, we systematically investigated the electronic and electrochemical performance of pristine and endohedral doped (O and Se) Ge_12_C_12_ and Si_12_C_12_ nanocages as a prospective negative electrode for lithium-ion batteries using high-level density functional theory at the DFT/B3LYP-GD3(BJ)/6-311 + G(d, p)/GEN/LanL2DZ level of theory. Key findings from frontier molecular orbital (FMO) and density of states (DOS) revealed that endohedral doping of the studied nanocages with O and Se tremendously enhances their electrical conductivity. Furthermore, the pristine Si_12_C_12_ nanocage brilliantly exhibited the highest V_cell_ (1.49 V) and theoretical capacity (668.42 mAh g^− 1^) among the investigated nanocages and, hence, the most suitable negative electrode material for lithium-ion batteries. Moreover, we utilized four machine learning regression algorithms, namely, Linear, Lasso, Ridge, and ElasticNet regression, to predict the V_cell_ of the nanocages obtained from DFT simulation, achieving R^2^ scores close to 1 (R^2^ = 0.99) and lower RMSE values (RMSE < 0.05). Among the regression algorithms, Lasso regression demonstrated the best performance in predicting the V_cell_ of the nanocages, owing to its L1 regularization technique.

**Supplementary Information:**

The online version contains supplementary material available at 10.1038/s41598-024-77150-x.

## Introduction

Lithium-ion batteries have garnered tremendous momentum in the field of energy storage and conversion devices due to their vast application in several energy-intensive devices, such as battery electric vehicles (BEVs), plug-in hybrid electric vehicles (PHEVs), electric bikes (e-bikes), solar cell batteries, computer electronics, and portable electronic devices^1–3^. The most intriguing feature of the global use of lithium-ion batteries in these electrical devices is their impressive energy density and long cycle life^4^. However, the ubiquitous use of graphite as an anode posed weak intercalation of Li, affecting its energy density for long-duration applications such as battery electric vehicles (BEVs), which constitute “range anxiety” for its users^5,6^. Hence, there is a sense of urgency to develop efficient and innovative anode material to navigate these challenges.

Recently, the scientific community has upgraded their wisdom in the utilization of 2D nanostructured materials, such as nanotubes^7^, graphene^8^, fullerenes^9^, and nanocones^10^ as anode material in rechargeable metal-ion batteries. Remarkable progress, such as doping^11^, functionalization^12^, and hydrogenation^13^ of several nanostructured materials have been employed to enhance their performance as negative electrode material in metal-ion batteries. Several literature studies have been conducted experimentally and theoretically to validate the deployment of nanostructured materials as anode material in lithium-ion batteries. Experimentally, Hu et al. synthesized porous carbon material with high storage capacity as negative electrode material in LiBs^14^. Zhang et al. experimentally investigated the utilization of V@MoS_2_ nanosheet as anode material in lithium-ion batteries. Their result revealed that the V@MoS_2_ nanosheet demonstrated a reversible charge capacity of 1047 mAh g^− 1^ with 700 cycles^15^.

More interestingly, with the advancement of computational approaches such as density functional theory, many scholars have scientifically analyzed and designed several nanostructured materials for functional application in lithium-ion batteries. For instance, Bagheri et al. utilized the DFT method to hydrogenate C_24_ fullerene as an efficient anode material for lithium-ion batteries. Key findings disclosed that hydrogenation of the C_24_ fullerene depicted an impressive storage capacity of 1.7 V and there was no manifestation of hindrance by the hydrogenation for Li-ion diffusion^16^. Hashemizadeh and coworkers theoretically scrutinized the fruitfulness of C_32_ and B_16_N_16_ nanocages as anode candidates in Li, K, and Na ion batteries. They reported the C_32_ and B_16_N_16_ nanocages explicated cell voltage of 1.03 and 1.18 V, respectively^17^. Using the DFT approach, Chen et al. structurally engineered C_24_, Si_24_, B_21_N_21_, and Al_21_P_21_ nanocages as anode material in Li, K, and Na ion batteries using the DFT approach. Their finding underscored that the studied C_24_, Si_24_, B_21_N_21_, and Al_21_P_21_ nanocages disclosed respective cell voltages of 0.88, 1.01, 1.24, and 1.43 V^18^. Kosar et al. recently applied the DFT method to ascertain the storage capacity of F^–^ encapsulation in Mg_12_O_12_ and Zn_12_O_12_ nanocages as negative electrodes in lithium-ion batteries. They asserted that encapsulation of F anion in Mg_12_O_12_ unveiled the highest cell voltage of 4.05 V and, thus, the most appropriate anode storage material for lithium-ion batteries^19^.

Motivated by these research articles, we proposed novel nanostructured materials as promising anode storage materials for lithium-ion batteries. Herein, we systematically investigated and assessed the electronic properties and storage capacity of pristine and endohedral doped (O and Se) Ge_12_C_12_ and Si_12_C_12_ nanocages as anode material in LiBs by employing advanced quantum mechanical approach at DFT/B3LYP-GD3(BJ)/6-311 + G(d, p)/GEN/LanL2DZ level of theory. In this report, frontier molecular orbital (FMO) analysis and density of states (DOS) were utilized to comprehensively understand the studied nanocages’ electronic properties. Adsorption energies of Li atom and Li^+^ cation on the titled nanocages were equally predicted to give insight into their application as anode material for lithium-ion batteries. Machine learning algorithms were used to predict the V_cell_ of the nanocages from DFT datasets. Based on these objectives, we attempt to scientifically address these fundamental research questions: {1} How does endohedral doping of O and Se affect the kinetic stability, chemical reactivity, and electrical conductivity of the studied nanocages? {2} How sensitive are Li atom and Li^+^ cation adsorption on the nanocages towards their energy gap? {3} How does the strength of the Li atom and Li^+^ cation interaction on the nanocages affect their cell voltage? {4} Which of these studied nanocages exhibited better cell voltage among the pristine and endohedral doped nanocages for potential application as anode material for lithium-ion batteries? {5} How accurate are the machine learning algorithms in predicting the cell voltage of the studied nanocages, and which of the algorithms performed better?

## Methodology

### Computational details

In this study, all relaxed geometry calculations were performed using Gaussian 16^20^ by employing DFT/B3LYP-GD3(BJ)/6-311 + G(d, p)/GEN/LanL2DZ level of theory. The B3LYP-GD3(BJ) exchange-correlation functional comprising of the D3 version of Grimme’s dispersion correction and Becke-Johnson damping correction was employed for this study as it accurately unveils weak interactions during adsorption on nanostructures as found in several literature and benchmark studies^19,21–23^, . The effective core potential and double polarization basis set (LanL2DZ) was assigned to the Ge, Se, and Si atoms. The combination of this basis set with an all-electron-basis set for non-transition or non-metal atoms has been demonstrated in several studies to be reasonable in revealing various molecular properties of systems^24,25^. Lighter atoms, such as oxygen, carbon, and lithium atoms, were assigned the 6-311 + G(d, p) basis set with the inclusion of the GEN keyword to specify a mixed basis set. Crucially, frequency calculation was estimated to ensure that all the investigated structures attained a minimum potential energy surface, and this was established by zero imaginary frequency. Furthermore, kinetic stability and chemical reactivity of the pristine, endohedral doped, and Li/Li^+^ adsorbed nanocages were delved into by performing frontier molecular orbital (FMO) analysis. The density of states (DOS) of the pristine, endohedral doped, and Li/Li^+^ adsorbed nanocages was evaluated using GaussSum software^26^. The relationship between electrical conductivity and energy gap is well-represented in Eq. (1).

1 $${\upsigma \upalpha }{\text{ exp}}\left( { - {\text{E}}_{{\text{g}}} /{\text{2kT}}} \right)$$

where σ represents electrical conductivity, E_g_ demonstrates the energy gap, k is the Boltzmann constant, and T depicts temperature.

Thermodynamics properties such as standard enthalpy of formation and standard Gibbs free energy of formation of the studied nanocages at 298.15 K and 1 atm were calculated using Eqs. (2) and (3).

2 $$\Delta {\text{H}}_{{\text{f}}}^{ \circ } = \Delta {\text{H}}_{{{\text{f}}({\text{Li}}/{\text{Li}} + @{\text{nanocage}})}}^{ \circ } {-}\Delta {\text{H}}_{{{\text{f}}({\text{nanocage}})}}^{ \circ } {-}\Delta {\text{H}}_{{{\text{f}}({\text{Li}}/{\text{Li}} + )}}^{ \circ }$$

3 $$\Delta {\text{G}}_{{\text{f}}}^{ \circ } = \Delta {\text{G}}_{{{\text{f}}({\text{Li}}/{\text{Li}} + @{\text{nanocage}})}}^{ \circ } {-}\Delta {\text{G}}_{{{\text{f}}({\text{nanocage}})}}^{ \circ } {-}\Delta {\text{G}}_{{{\text{f}}({\text{Li}}/{\text{Li}} + )}}^{ \circ }$$

$\Delta {\text{H}}_{{\text{f\, (Li/Li}}+@{\text{nanocage)}}}^{ \circ }$ and $\Delta {\text{G}}_{{\text{f\, (Li/Li}}+@{\text{nanocage)}}}^{ \circ }$ describe the standard enthalpy of formation and standard Gibbs free energy of formation of the Li/Li^+^ adsorbed nanocages, respectively, $\Delta {\text{H}}_{{\text{f\, (nanocage)}}}^{ \circ }$ and $\Delta {\text{G}}_{{\text{f\, (nanocage)}}}^{ \circ }$ defines the standard enthalpy of formation and standard Gibbs free energy of formation of the bare nanocages, correspondingly, $\Delta {\text{H}}_{{\text{f\, (Li/Li)}}+}^{ \circ }$ and $\Delta {\text{G}}_{{\text{f\, (Li/Li)}}+}^{ \circ }$ reveals the standard enthalpy of formation and standard Gibbs free energy of formation of Li/Li^+^, respectively.

Adsorption energies of Li^+^ cation and Li atom on the pristine and endohedral doped nanocages were computed using Eqs. (4) and (5), respectively.

4 $${\text{E}}_{{{\text{ads}}}} = {\text{ E}}_{{{\text{cage}}/{\text{Li}} + }} {-}{\text{ E}}_{{{\text{cage}}}} {-}{\text{ E}}_{{{\text{Li}} + }} + \Delta {\text{E}}_{{({\text{BSSE}})}} + \Delta {\text{E}}_{{({\text{ZPE}})}}$$

5 $${\text{E}}_{{{\text{ads}}}} = {\text{ E}}_{{{\text{cage}}/{\text{Li}}}} {-}{\text{ E}}_{{{\text{cage}}}} {-}{\text{ E}}_{{{\text{Li}}}} + \Delta {\text{E}}_{{({\text{BSSE}})}} + \Delta {\text{E}}_{{({\text{ZPE}})}}$$

E_cage/Li+_ and E_cage/Li_ depict the total energy of the Li^+^ cation and Li atom adsorbed nanocages; E_Li+_ and E_Li_ reflect the total energy of the Li^+^ cation and Li atom, respectively. The Basis set superposition error (BSSE) was estimated using the counterpoise method proposed by Boys and Bernardi as indicated in Eq. (6)^27^.

6 $$\:{\varDelta\:E}_{\left(BSSE\right)}={\varDelta\:E}_{cluster}-{\varDelta\:E}_{cage}^{cluster}-{\varDelta\:E}_{Li/Li+}^{cluster}$$

### Machine learning

Machine learning is widely used to predict material properties due to the robustness of various machine learning algorithms. Supervised machine learning algorithms such as regression and classification have gained recognition for their ability to make scientific predictions based on datasets from experimental or computational studies. For instance, researchers have used these algorithms to predict the electronic and adsorption properties of molecular systems^28,29^.

**Fig. 1:**
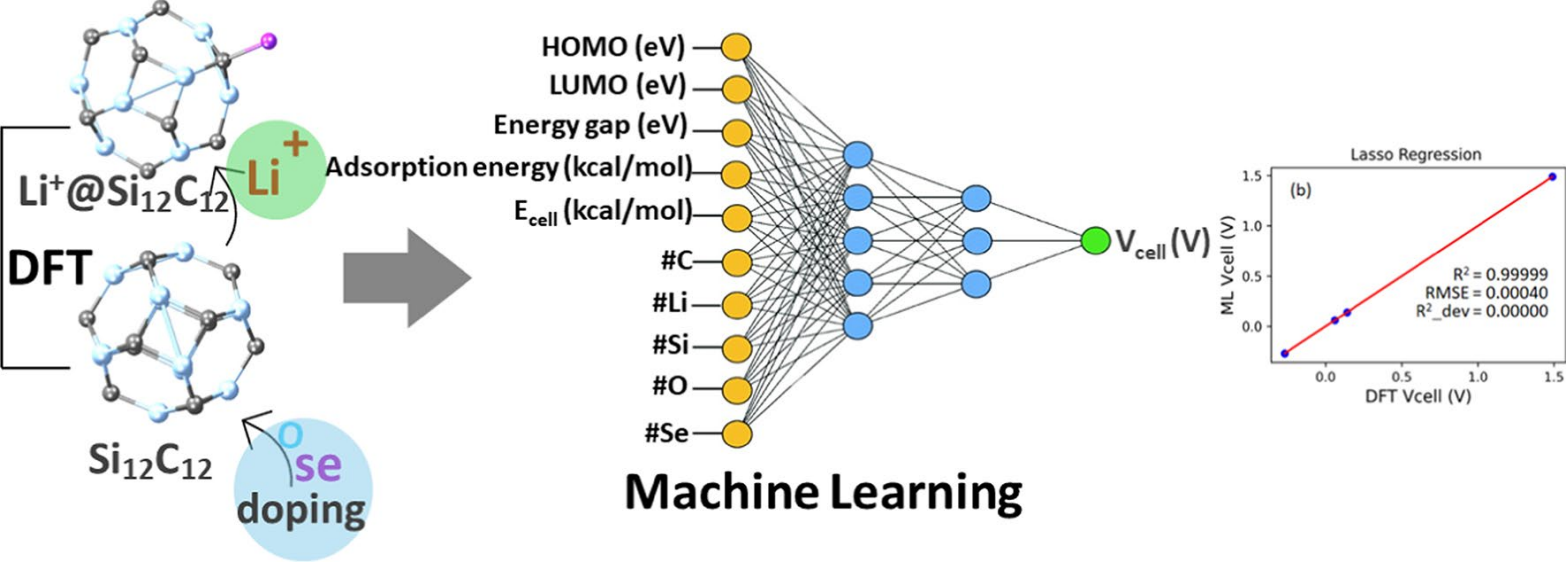
Graphical illustration from DFT modelling to machine learning prediction.

In this work, we employed four regression models (Linear, Ridge, Lasso, and ElasticNet) to predict the V_cell_ of our studied nanocages using datasets from DFT simulations. We selected these regression models because they can make accurate predictions using smaller datasets and are robust to overfitting. Therefore, we utilized a small dataset (containing six rows) from our DFT modeling, which included columns of the energy gap, ∆E_cell_, and V_cell_ of the pristine and endohedral doped nanocages for our machine learning predictions. Figure 1. shows the graphical illustration from DFT modelling to machine learning predictions. The subsequent sections will provide a detailed discussion of the regression models used in our machine-learning predictions.

#### Linear regression

Linear regression is a machine learning algorithm demonstrating a linear relationship between a dataset’s independent features and the dependent target variables. Depending on the number of input features used for prediction, it can be classified into simple and multiple linear regression. Simple linear regression predicts a target variable (y) from a single input feature (x), whereas multiple linear regression predicts a target variable from multiple input features.

In this research, we employed multiple linear regression to predict the V_cells_ of our studied nanocages as anode material in lithium-ion batteries. The input features used were the energy gap and ∆E_cell_ of the nanocages obtained from DFT modeling. The mathematical expression for multiple linear regression is given by Eq. (7).

7 $${\text{y}} ={\upbeta }0 + {\upbeta }{{1}} {\text{ x}} {{1}} + {{\upbeta 2}} {\text{ x}}{{2}} + \ldots + {\upbeta }{\text{n}} {\text{ x}} {\text{n}} + {\upvarepsilon }$$

y is the target variable, β0 represents the intercept, β1, β2, and βn signifies slope coefficients, x1, x2, and xn demonstrates the input features, and ε depicts the error term or residuals.

#### Lasso regression

Lasso regression, also known as Least Absolute Shrinkage and Selection Operator, is a regularization technique used in linear regression to mitigate overfitting. The main goal of regularization in Lasso regression is to enhance the model’s ability to new data. This is accomplished by introducing a penalty term to the cost function using the L1 regularization method, which is proportionate to the absolute value of the coefficients^30^. This method effectively performs feature selection by setting coefficients of unimportant features to zero. The parameter alpha (α) dictates the strength of regularization in Lasso regression, with a higher value of α resulting in more coefficients being set to zero. Lasso regression can be estimated using Eq. (8).

8 $${\text{y }} = \upbeta {\text{0 }} + \upbeta {\text{1x1 }} + \upbeta {\text{2x2 }} + \ldots + \upbeta {\text{nxn }} + \varepsilon + \alpha \left| {\upbeta {\text{1, }}\upbeta {\text{2}} \ldots \upbeta {\text{n}}} \right|$$

y is the target variable, β0 represents the intercept, β1, β2, and βn signifies slope coefficients, x1, x2, and xn demonstrates independent variables, ε depicts the error term or residuals, and α indicates the regularization parameter.

#### Ridge regression

Ridge regression is another regularization technique used to penalize cost function in linear regression. Similar to Lasso regression, Ridge regression combats overfitting by generating robust solutions for new, unseen data with smaller coefficients. It employs the L2 regularization method, which penalizes the cost function in proportion to the square of the coefficients. This method effectively addresses issues such as multicollinearity and overfitting by shrinking the coefficients toward zero without actually reaching zero^31^. Consequently, it enhances model generalization in the presence of new data by reducing the magnitude of the model coefficients. The strength of the regularization in Ridge regression is determined by the alpha (α) parameter. A high value of α demonstrates a stronger penalty, shrinking the coefficients aggressively. The key difference between L1 and L2 regularization in handling multicollinearity is that the coefficients are set to zero and shrunk towards zero, respectively. Statistically, Ridge regression is evaluated using Eq. (9).

9 $${\text{y}} = \upbeta 0 + \upbeta {\text{1x1}} + \upbeta {\text{2x2}} + \ldots + \upbeta {\text{nxn}} + \upvarepsilon + \upalpha \left( {\upbeta {\text{1}},\upbeta {\text{2}} \ldots \upbeta {\text{n}}} \right)^{{\text{2}}}$$

y is the dependent variable, β0 represents the intercept, β1, β2, and βn signifies slope coefficients, x1, x2, and xn demonstrates independent variables, ε depicts the error term or residuals, and α indicates the regularization parameter.

#### ElasticNet regression

ElasticNet regression is a valuable regularization method for mitigating overfitting in linear regression models. It combines L1 and L2 regularization techniques to penalize the cost function^32^. By striking a balance between Lasso and Ridge regressions, ElasticNet regression effectively shrinks the coefficients towards zero, rendering unimportant features as zero. The model’s regularization strength is governed by the alpha (α) parameter, and the l1_ratio parameter, representing the ratio of L1 to L2 regularization. As α and l1_ratio increase, the model transitions from Ridge to Lasso regression, effectively reducing overfitting. The implementation of ElasticNet regression can be mathematically expressed using Eq. (10).

10 $${\text{y }} = \upbeta 0 + \upbeta {\text{1x1}} + \upbeta {\text{2x2}} + \ldots + \upbeta {\text{nxn}} + \upvarepsilon + \alpha \left( {\upbeta {\text{1}},\,\upbeta {\text{2}} \ldots \,\upbeta {\text{n}}} \right)^{{\text{2}}} + \alpha \left| {\upbeta {\text{1}},\,\upbeta {\text{2}} \ldots \,\upbeta {\text{n}}} \right|$$

y is the dependent variable, β0 represents the intercept, β1, β2, and βn signifies slope coefficients, x1, x2, and xn demonstrates independent variables, ε depicts the error term or residuals, and α indicates the regularization parameter.

#### Model training, validation, and evaluation

To optimize the performance of our regression models on our small-sized datasets (six rows), we split our datasets (X and Y variables) into training (X_train and Y_train) and testing (X_test and Y_test) datasets with an 80%:20% ratio, using random_state of 18. Subsequently, we performed cross-validation on our regression models to assess their general performance on unseen test datasets. For this reason, we utilized the k-fold cross-validation method to evaluate cross-validation on our training datasets by splitting them into subsets known as folds.

Due to the small size of our datasets, we split our training datasets (X_train and Y_train) into two k-folds using n_splits = 2 and iterated twice on different subsets to ensure excellent performance of regression models. The k-1 fold (X_train_fold and Y_train_fold) was used to train the regression models, while the models were evaluated using the remaining one-fold (X_test_fold and Y_test_fold). Additionally, hyperparameter tuning was carried out on the regularized regression models to enhance the generalization of the models during cross-validation. Moreover, we utilized cross_val_score metrics to evaluate the performance of the k-fold cross-validation technique using R^2^ and root mean squared error (RMSE) as the scoring functions. The estimated R^2^ and RMSE can be referenced in ref^33^. The machine learning code associated with this prediction can be accessed via the GitHub repository (link in the data availability statement).

## Results and discussion

### Geometry, structural, and thermodynamics properties

**Fig. 2:**
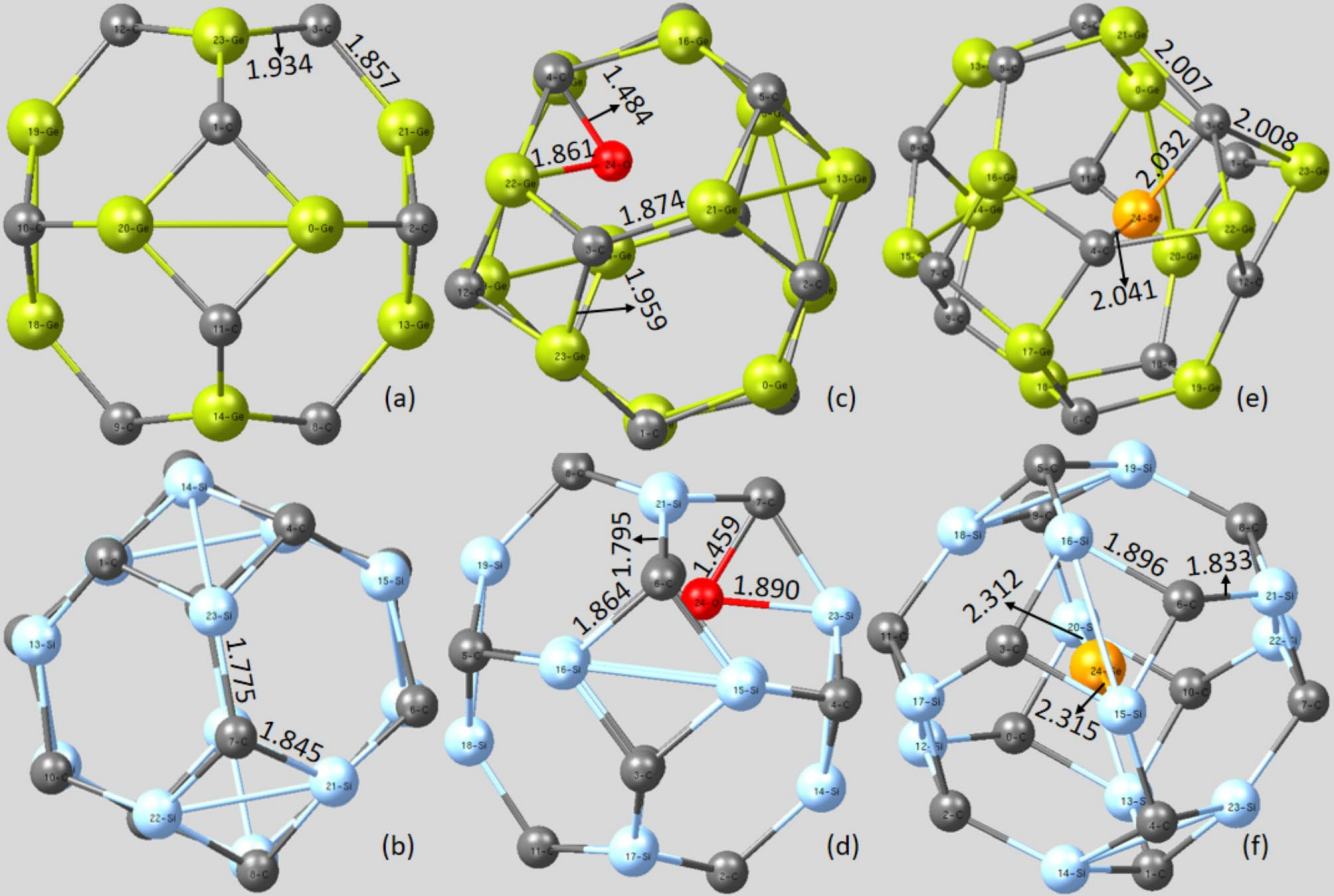
Relaxed geometry of the pristine and endohedral doped nanocages without Li/Li^+^ adsorption. (**a**) Ge_12_C_12_ (**b**) Si_12_C_12_ (**c**) O-Ge_12_C_12_ (**d**) O-Si_12_C_12_ (**e**) Se-Ge_12_C_12_ (**f**) Se-Si_12_C_12_.

**Fig. 3:**
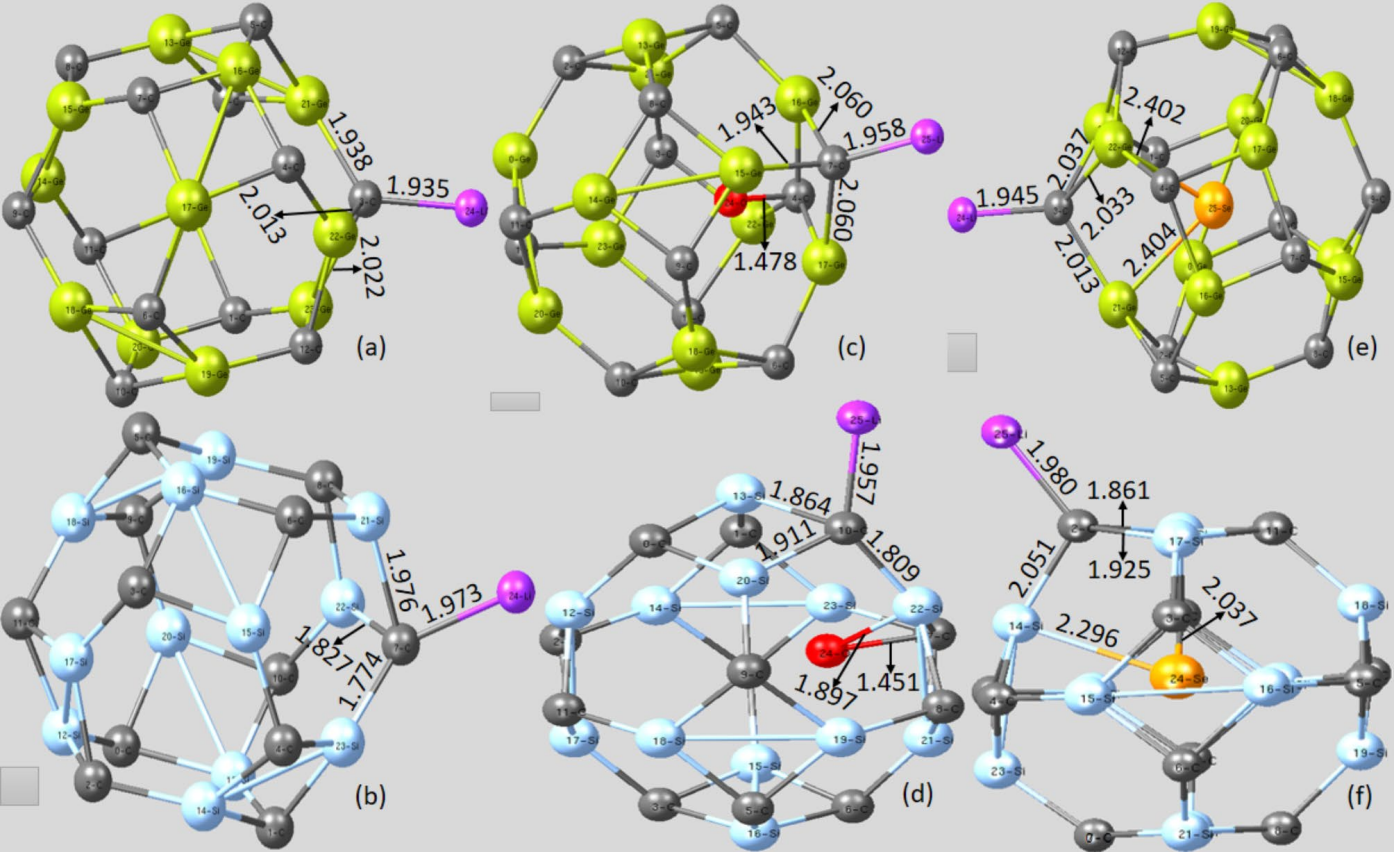
Relaxed geometry of the pristine and endohedral doped nanocages with Li adsorption. (**a**) Li@Ge_12_C_12_ (**b**) Li@Si_12_C_12_ (**c**) Li@O-Ge_12_C_12_ (**d**) Li@O-Si_12_C_12_ (**e**) Li@Se-Ge_12_C_12_ (**f**) Li@Se-Si_12_C_12_.

**Fig. 4:**
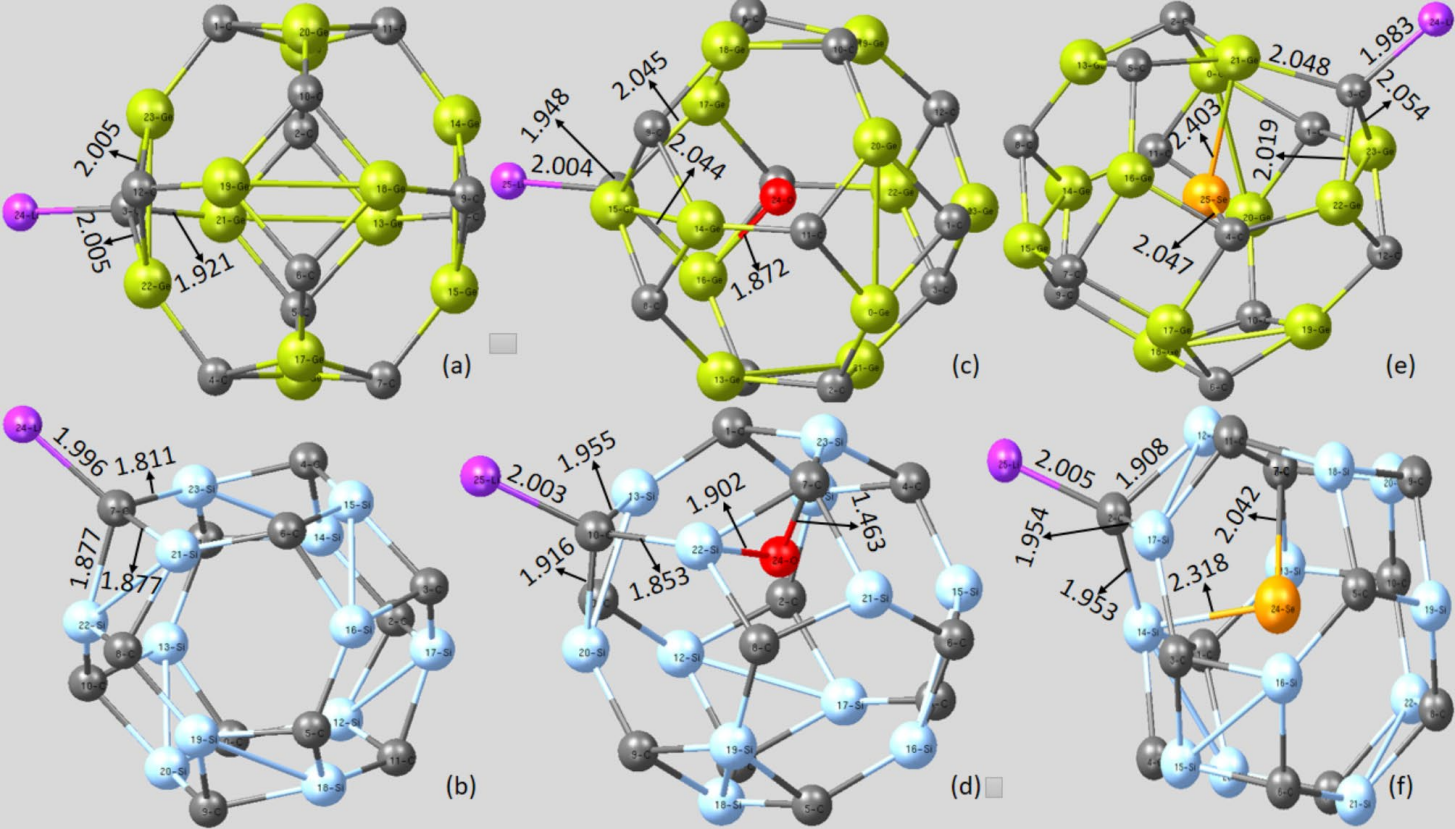
Relaxed geometry of the pristine and endohedral doped nanocages with Li^+^ adsorption. (**a**) Li^+^@Ge_12_C_12_ (**b**) Li^+^@Si_12_C_12_ (**c**) Li^+^@O-Ge_12_C_12_ (**d**) Li^+^@O-Si_12_C_12_ (**e**) Li^+^@Se-Ge_12_C_12_ (**f**) Li^+^@Se-Si_12_C_12_.

Optimized geometry of the modeled Ge_12_C_12_ and Si_12_C_12_ nanocages were painstakingly characterized at DFT/B3LYP-GD3(BJ)/6-311 + G(d, p)/GEN/LanL2DZ level of theory. The investigated Ge_12_C_12_ and Si_12_C_12_ nanocages constitute eight hexagonal and six tetragonal rings, as shown in Figs. 2, 3 and 4. Notably, two sets of Ge-C and Si-C bonds were evident in the studied cages: the bond between a hexagonal and a tetragonal ring (**d**_**6,4**_**)** and between two hexagonal rings (**d**_**6,6**_). This analysis selected a d6,4 bond and d6,6 bond for the pristine and endohedral doped nanocages without Li/Li^+^ adsorption due to their similar bond lengths. In the case of Li/Li^+^ adsorbed nanocages, two **d**_**6,4**_ bonds and a **d**_**6,6**_ bond closer to the adsorbed Li atom and Li^+^ cation were represented as they experienced a greater charge transfer effect from the Li atom and Li^+^ cation.

For the pristine Ge_12_C_12_ nanocage, the **d**_**6,4**_ and **d**_**6,6**_ bonds were on Ge_23_-C_3_ and Ge_21_-C_3_ with corresponding bond lengths of 1.934 Å and 1.857 Å. Similarly, the **d**_**6,4**_ and **d**_**6,6**_ bonds for the pristine Si_12_C_12_ nanocage were noticed on Si_21_-C_7_ and Si_23_-C_8_, possessing specific bond lengths of 1.845 Å and 1.775 Å. The reported Ge-C and Si-C bond lengths are comparable to those in ref^34,35^.

Upon endohedral doping of the Ge_12_C_12_ and Si_12_C_12_ nanocage with O and Se, the selected **d**_**6,4**_ and **d**_**6,6**_ bonds in the Ge_12_C_12_ and Si_12_C_12_ nanocage were elongated. This observed phenomenon can be related to the electronegative nature of the O and Se anions, which tend to spread their negative charge towards the studied cages, resulting in polarization of the Ge-C and Si-C bonds. In the O-Ge12C12 endohedral doped nanocage context, the selected **d**_**6,4**_ and **d**_**6,6**_ bonds were on Ge_23_-C_3_ and Ge_21_-C_3_ with bond distances of 1.959 Å and 1.875 Å, respectively. During charge distribution toward the surface of the nanocage, the endohedral doped O anion bonded with a Ge and C atom, forming a Ge-O-C bond.

Concerning O-Si_12_C_12_ endohedral doped nanocage, the selected **d**_**6,4**_ and **d**_**6,6**_ bonds were seen on Si_16_-C_6_ and Si_21_-C_6_ with respective bond lengths of 1.847 Å and 1.795 Å. Additionally, the endohedral doped O anion bonded with a Si and C atom of the nanocage, yielding a Si-O-C bond. The Si-O and C-O bonds possessed respective bond distances of 1.890 Å and 1.459 Å. The formation of Si-O can be attributed to the strong affinity of the Si atom to attack the highly electronegative O anion by pulling the valence electron in the p-orbital of the O anion into its vacant d-orbital, forming a stable Si-O bond with partial π character. Even though the Si-O bond is longer (1.890 Å) than the C-O single bond (1.459 Å) formed, the Si-O is stronger due to its partial π character. At the same time, the C-O single bond is weaker as it depicts a pure covalent bond, which is also confirmed in ref^36–38^.

Moving to the Se-Ge_12_C_12_ endohedral nanocage, the selected **d**_**6,4**_ and **d**_**6,6**_ bonds were noted on Ge_23_-C_3_ and Ge_21_-C_3_ bonds with individual bond lengths of 2.008 Å and 2.007 Å. Notably, the endohedral doped Se tends to stabilize itself by forming two nonpolar covalent Se-C bonds due to the similar electronegativity of Se and C (2.55) on the Pauling electronegativity scale^39^. The observed Se-C bonds were visible on Se_24_-C_3_ and Se_24_-C_4_ bonds with respective bond distances of 2.032 Å and 2.041 Å. Next, the selected **d**_**6,4**_ and **d**_**6,6**_ bonds available on the Se-Si_12_C_12_ endohedral doped nanocage were demonstrated by Si_16_-C_6_ and Si_21_-C_6_ bonds with specific bond lengths of 1.896 Å and 1.833 Å. Excitingly, the Se anion formed a Si-Se-Si bond within the Se-Si_12_C_12_ endohedral doped nanocage, having an average Si-Se bond distance of 2.314 Å.

Initially, adsorption of Li/Li^+^ on the pristine and endohedral doped nanocages was modelled on different sites of the nanocages: on the centre of the hexagon and tetragon, on the **d**_**6,4**_ and **d**_**6,6**_ bonds, on top of Ge, Si, and C atoms. However, post-optimization of the Li/Li^+^ adsorbed pristine- and endohedral doped nanocages showcased that the Li atom and Li^+^ cation preferentially bind to the C atom of the nanocages, attributed to the higher electronegativity of the C atom juxtaposed to the Ge and Si atoms making the highly electropositive Li/Li^+^ to adsorb on it. The influence of charge transfer on the bond from the Li atom and Li^+^ cation on the pristine and endohedral doped nanocages post-adsorption was also considered.

Table 1. revealed that Li/Li^+^ adsorption on the pristine Ge_12_C_12_ and Si_12_C_12_ nanocages elongated the selected **d**_**6,4**_ and **d**_**6,6**_ bonds with maximum charge transfer effect compared to the investigated pristine nanocages without Li/Li^+^ adsorption. For pristine Li@Ge_12_C_12_ nanocage, the selected two **d**_**6,4**_ bonds on Ge_22_-C_3_ and Ge_23_-C_3_ elongated from 1.934 to 2.013 Å and 2.022 Å, respectively, while the observed **d**_**6,6**_ bond on Ge_21_-C_3_ increased from 1.857 to 1.938 Å. Regarding Li^+^@Ge_12_C_12_ nanocage, **d**_**6,4**_ bonds represented by Ge_22_-C_3_ and Ge_23_-C_3_ increased remarkably from 1.934 to 2.005 Å and 2.005 Å, respectively, while the **d**_**6,6**_ bond on Ge_21_-C_3_ incremented from 1.857 to 1.921 Å.

Assessing the two **d**_**6,4**_ bonds present on Li@Si_12_C_12_ nanocage revealed that the Si_21_-C_7_ and Si_22_-C_7_ bonds are 1.976 Å and 1.827 Å, respectively, while there is a slight variation of the **d**_**6,6**_ bond on Si_23_-C_7_ from 1.775 to 1.774 Å. Visualizing the effects of charge transfer on the bonds of Li^+^@Si_12_C_12_ nanocage, its **d**_**6,4**_ bonds on Si_21_-C_7_ and Si_22_-C_7_ were both stretched from 1.845 to 1.877 Å, while the **d**_**6,6**_ bond was extended from 1.775 to 1.811 Å.

Going further, we also investigated the effect of charge transfer upon the adsorption of Li atom and Li^+^ cation on the endohedral doped (O and Se) Ge_12_C_12_ and Si_12_C_12_ nanocages. As depicted in Table 2., it is obvious that adsorption of Li atom and Li^+^ cation enhances the bond lengths of the two **d**_**6,4**_ bonds and **d**_**6,6**_ bond in the endohedral doped Ge_12_C_12_ and Si_12_C_12_ nanocages, reflecting polarization of nanocages bonds due to sufficient charge transfer from the O and Se endohedral dopants towards the surface of the cage, as well as charge transfer from the adsorbed Li atom and Li^+^ cation to the nanocage.

Analyzing the effect of charge transfer on the Li@O-Ge_12_C_12_ nanocage disclosed that the two **d**_**6,4**_ bonds on Ge_16_-C_7_ and Ge_17_-C_7_ were both outstretched from 1.959 to 2.060 Å, while the **d**_**6,6**_ bond Ge_15_-C_7_ was lengthened from 1.874 to 1.943 Å. Interestingly, a C-O single bond was formed between the endohedral doped O and C atom of the Li@O-Ge_12_C_12_ nanocage on C_4_-O_24_ with a bond length of 1.478 Å. This observed bond can be related to the electrophilic nature of the C atom, which tends to pull lone pair electrons of oxygen, forming a covalent bond afterwards. In the same way, the two **d**_**6,4**_ bonds on Li^+^@O-Ge_12_C_12_ nanocage with bond labeling of Ge_16_-C_7_ and Ge_17_-C_7_ were significantly extended from 1.959 to 2.044 Å and 2.045 Å, correspondingly, while the **d**_**6,6**_ bond on Ge_15_-C_7_ elongated from 1.874 to 1.948 Å. Notably, an endohedral doped Ge_16_-O_24_ bond was observed in the Li^+^@O-Ge_12_C_12_ nanocage with a bond distance of 1.872 Å.

For the Li@O-Si_12_C_12_ nanocage, the **d**_**6,4**_ bonds on Si_13_-C_10_ and Si_20_-C_10_ extended from 1.847 to 1.864 Å and 1.911 Å, respectively, while the **d**_**6,6**_ bond on Si_22_-C_10_ dramatically increased from 1.795 to 1.809 Å. Consequently, an endohedral doped Si-O-C bond was evident on the Si_23_-O_24_-C_7_ bond. This observed Si-O-C bond is unstable due to the polarity of Si-O present in the bond. Diving deep into the charge transfer effect on Li^+^@O-Si_12_C_12_ nanocage, the selected two **d**_**6,4**_ bonds on Si_13_-C_10_ and Si_20_-C_10_ augmented from 1.847 to 1.955 Å and 1.916 Å, respectively, while the **d**_**6,6**_ bond on Si_22_-C_10_ enlarged from 1.795 to 1.853 Å. Additionally, a Si-O-C bond was obvious in the Li^+^@O-Si_12_C_12_ nanocage at the Si_23_-O_24_-C_7_ bond.

In regards to Li@Se-Ge_12_C_12_ nanocage, the two **d**_**6,4**_ bonds on Ge_22_-C_3_ and Ge_23_-C_3_ elongated from 2.008 to 2.033 Å and 2.037 Å, respectively, while the **d**_**6,6**_ bond positioned on Ge_21_-C_3_ protracted from 2.007 to 2.013 Å. Interestingly, a Ge-Se-Ge endohedral bond was noted on Ge_21_-Se_25_-Ge_22_ with an average Ge-Se bond length of 2.403 Å. Correspondingly, the two **d**_**6,4**_ bonds on Li^+^@Se-Ge_12_C_12_ nanocage were situated on Ge_22_-C_3_ and Ge_23_-C_3_ extended from 2.008 to 2.019 Å and 2.054 Å, respectively. In contrast, its **d**_**6,6**_ bond on Ge_21_-C_3_ stretched from 2.007 to 2.048 Å. An endohedral Ge-Se-C bond was formed on the Ge_21_-Se_25_-C_4_, exhibiting Ge-Se and Se-C bond distances of 2.403 Å and 2.047 Å, respectively.

Moreover, bond polarization on Li@Se-Si_12_C_12_ nanocage unveiled that the **d**_**6,4**_ bonds decreased slightly from 1.896 to 1.861 Å for Si_12_-C_2_ and enlarged from 1.896 to 1.925 Å for Si_17_-C_2_ bond. Meanwhile, its **d**_**6,6**_ bond on Si_14_-C_2_ expanded from 1.833 to 2.051 Å. In the same vein, the two **d**_**6,4**_ bonds on the Li^+^@Se-Si_12_C_12_ nanocage which were noted on Si_12_-C_2_ and Si_17_-C_2_ bonds prolonged from 1.846 to 1.908 Å and 1.896 to 1.954 Å, correspondingly, while the **d**_**6,6**_ bond on Si_14_-C_2_ increased sharply from 1.833 to 1.953 Å. Endohedral Si-Se-C bonds were present in Li@Se-Si_12_C_12_ and Li^+^@Se-Si_12_C_12_ nanocages.

Lastly, thermodynamic properties such as standard enthalpy of formation (∆H_f_^⁰^) and standard Gibbs free energy of formation (∆G_f_^⁰^) of the nanocages were explicitly calculated to confirm their stability. As seen in Table S1**.**, Li@Si_12_C_12_ and Li^+^@Si_12_C_12_ nanocages revealed the least ∆H_f_^⁰^ and ∆G_f_^⁰^ values, indicating that the pristine Si_12_C_12_ nanocage is the most thermodynamically stable nanostructure for Li atom and Li^+^ cation adsorption.

**Table 1:** Structural properties of pristine Ge_12_C_12_ and Si_12_C_12_ nanocages; adsorption energy in kcal/mol (E_ads_), bond length of adsorption (d_ads_), bond length between hexagonal and tetragonal rings (d_64_), bond length between two hexagonal rings (d_66_).

Structure	E_ads_	d_ads_	(Å)	d_64_	(Å)	d_66_	(Å)
Ge_12_C_12_	–	–	–	Ge_23_-C_3_	1.934	Ge_21_-C_3_	1.857
Li@Ge_12_C_12_	-16.60	Li_24_-C_3_	1.935	Ge_22_-C_3_	2.013	Ge_21_-C_3_	1.938
–	–	–	–	Ge_23_-C_3_	2.022	–	–
Li^+^@Ge_12_C_12_	-17.99	Li_24_-C_3_	1.990	Ge_22_-C_3_	2.005	Ge_21_-C_3_	1.921
–	–	–	–	Ge_23_-C_3_	2.005	–	–
Si_12_C_12_	–	–	–	Si_21_-C_7_	1.845	Si_23_-C_7_	1.775
Li@Si_12_C_12_	-37.36	Li_24_-C_7_	1.973	Si_21_-C_7_	1.976	Si_23_-C_7_	1.774
–	–	–	–	Si_22_-C_7_	1.827		
Li^+^@Si_12_C_12_	-71.72	Li_24_-C_7_	1.996	Si_21_-C_7_	1.877	Si_23_-C_7_	1.811
–	–	–	–	Si_22_-C_7_	1.877	–	–

**Table 2:** Structural properties of endohedral doped (O and Se) Ge_12_C_12_ and Si_12_C_12_ nanocages; adsorption energy in kcal/mol (E_ads_), bond length of adsorption (d_ads_), bond length between hexagonal and tetragonal rings (d_64_), bond length between two hexagonal rings (d_66_), bond length formed by endohedral dopants (d_dope_).

Structure	E_ads_	d_ads_	(Å )	d_6,4_	(Å)	d_6,6_	(Å)	d_dope_	(Å)
O-Ge_12_C_12_	–	–	–	Ge_23_-C_3_	1.959	Ge_21_-C_3_	1.874	Ge_12_-O_24_-C_4_	–
–	–	–	–	–	–	–	–	Ge_12_-O_24_	1.861
–	–	–	–	–	–	–	–	C_4_-O_24_	1.484
Li@O-Ge_12_C_12_	-62.03	Li_25_-C_7_	1.958	Ge_16_-C_7_	2.060	Ge_15_-C_7_	1.943	C_4_-O_24_	1.478
–	–	–	–	Ge_17_-C_7_	2.060	–	–	–	–
Li^+^@O-Ge_12_C_12_	-55.81	Li_25_-C_7_	2.004	Ge_16_-C_7_	2.044	Ge_15_-C_7_	1.948	Ge_16_-O_24_	1.872
–		–	–	Ge_17_-C_7_	2.045	–	–	–	–
O-Si_12_C_12_	–	–	–	Si_16_-C_6_	1.847	Si_21_-C_6_	1.795	Si_23_-O_24_-C_7_	–
–	–	–	–	–	–	–	–	Si_23_-O_24_	1.890
–	–	–	–	–	–	–	–	C_7_-O_24_	1.459
Li@O-Si_12_C_12_	-53.50	Li_25_-C_10_	1.957	Si_13_-C_10_	1.864	Si_22_-C_10_	1.809	Si_23_-O_24_-C_7_	-
				S_20_-C_10_	1.911	–	–	Si_23_-O_24_	1.897
–	–	–	–	–	–		–	C_7_-O_24_	1.451
Li^+^@O-Si_12_C_12_	-56.73	Li_25_-C_10_	2.003	Si_13_-C_10_	1.955	Si_22_-C_10_	1.853	Si_23_-O_24_-C_7_	–
–	–	–	–	S_20_-C_10_	1.916	–	–	Si_23_-O_24_	1.902
–	–	–	–	–	–	–	–	C_7_-O_24_	1.463
Se-Ge_12_C_12_	–	–	–	Ge_23_–C_3_	2.008	Ge_21_-C_3_	2.007	C_3_-Se_24_-C_4_	–
–	–	–	–	–	–	–	–	Se_24_-C_3_	2.032
–	–	–	–	–	–	–	–	Se_24_-C_4_	2.041
Li@Se-Ge_12_C_12_	-19.60	Li_24_-C_3_	1.945	Ge_22_-C_3_	2.033	Ge_21_-C_3_	2.013	Ge_21_-Se_25_-Ge_22_	-
–	–	–	–	Ge_23_-C_3_	2.037	–	–	Ge_21_-Se_25_	2.404
–	–	–	–	–	–	–	–	Ge_22_-Se_25_	2.402
Li^+^@Se-Ge_12_C_12_	-22.14	Li_24_-C_3_	1.983	Ge_22_-C_3_	2.019	Ge_21_-C_3_	2.048	Ge_21_-Se_25_-C_4_	–
–	–	–	–	Ge_23_-C_3_	2.054	–	–	Ge_21_-Se_25_	2.403
–	–	–	–	–	–	–	–	Se_25_-C_4_	2.047
Se-Si_12_C_12_	–	–	–	Si_16_-C_6_	1.896	Si_21_-C_6_	1.833	Si_15_-Se_24_-Si_20_	–
–	–	–	–	–	–	–	–	Si_15_-Se_24_	2.315
–	–	–	–	–	–	–	–	Si_20_-Se_24_	2.312
Li@Se-Si_12_C_12_	-55.81	Li_25_-C_2_	1.980	Si_12_-C_2_	1.861	Si_14_-C_2_	2.051	Si_14_-Se_25_-C_7_	–
–	–	-	–	Si_17_-C_2_	1.925	–	-	Si_14_-Se_25_	2.296
–	–	–	–	–	–	–	–	Se_25_-C_7_	2.037
Li^+^@Se-Si_12_C_12_	-58.57	Li_25_-C_2_	2.005	Si_12_-C_2_	1.908	Si_14_-C_2_	1.953	Si_14_-Se_25_-C_7_	–
–	–	–	–	Si_17_-C_2_	1.954	–	–	Si_14_-Se_25_	2.318
–	–	–	–	–	–	–	–	Se_25_-C_7_	2.042

### Electronic properties

Frontier molecular orbital is a fundamental and quintessential quantum mechanical approach utilized to better understand compounds’ kinetic stability and chemical reactivity^40^. It also disclosed functional information on the electrical conductivity of chemical systems in the presence of an applied external electric field^41^. Generally, the highest occupied molecular orbital (HOMO) and the lowest unoccupied molecular orbital (LUMO) are key molecular orbitals that are accommodated in the frontier molecular orbital. The HOMO plays a vital role in the donation of electrons, while the acceptance of electrons is the key responsibility of the LUMO in the frontier molecular orbital. Noteworthy, the discrepancy between the HOMO and the LUMO is related to the energy gap. Specifically, the energy gap is chiefly responsible for unveiling crucial information on a chemical system’s kinetic stability, chemical reactivity, and electrical conductivity^42^. A chemical system depicting a high energy gap value is confined with more excellent stability and lesser reactivity. In contrast, a lower energy gap value indicates that the chemical system is less stable and highly prone to chemical reactivity.

In this regard, we probed the kinetic stability and chemical reactivity of the studied pristine, endohedral doped, and Li/Li^+^ adsorbed nanocages based on the insight of their energy gaps. The variations of the energy gaps of the simulated nanocages are visible in Tables 3 and 4. Table 3 shows that the pristine Ge_12_C_12_ nanocage brilliantly exhibited an energy gap (E_g_) value of 2.01 eV. However, upon adsorption of the Li atom on the pristine Ge_12_C_12_ nanocage, its E_g_ experienced a dramatic decrease to 1.68 eV with a -16.42% reduction in E_g_. Similarly, the adsorption of Li^+^ cation on the Ge_12_C_12_ pristine nanocage demonstrated a decline in its energy gap to 1.99 eV with a percentage in reduction of -1.00%. This persisting decrease in energy gap for the Li@Ge_12_C_12_ and Li^+^@Ge_12_C_12_ adsorbed nanocages compared to the pristine Ge_12_C_12_ nanocage is attributed to the accommodation of charge transfer from the adsorbed Li atom and Li^+^ cation, which tends to polarize the Ge-C bonds, resulting to their proneness to chemical attack easily. Comparably, Li@Ge_12_C_12_ adsorbed nanocage revealed a lower energy gap value than Li^+^@Ge_12_C_12_ adsorbed nanocage as a result of the presence of SOMO in its HOMO orbital, which can be given out easily for chemical reactivity than fully occupied HOMO orbital of the Li^+^@Ge_12_C_12_ adsorbed nanocage.

Scrutinizing the pristine Si_12_C_12_ nanocage, it displayed an E_g_ value of 2.72 eV. Analogously, the pristine Si_12_C_12_ nanocage manifested higher E_g_ (2.72 eV) than the pristine Ge_12_C_12_ nanocage (2.01 eV), indicating more stability than the latter. This observation can be related to the stability of the Si-C bond, with an approximate bond energy of 318 KJ/mol^43^, compared to the less stable Ge-C bond (238 KJ/mol)^44^. From this inception, it is prominent that the Si-C bond is less reactive and polarizable by a chemical reagent or applied external field than the less stable Ge-C bond.

Examining the influence of adsorption of Li atom and Li^+^ cation on pristine Si_12_C_12_ nanocage, it was noted that Li@Si_12_C_12_ adsorbed nanocage reduces the energy gap of the pristine Si_12_C_12_ nanocage from 2.72 to 1.69 eV, possessing − 1.10% reduction in the energy gap. This observed result can be related to the presence of SOMO in the HOMO, which can quickly release its single electron for chemical reactivity and electrical conductivity. On the contrary, upon adsorption of Li + cation on the pristine Si_12_C_12_ nanocage, its energy gap increased rapidly from 2.72 to 3.33 eV, with a 22.43% increment in its energy gap value. This result can be linked to the slight resistance of Li^+^ cation transferred charge to the pristine Si_12_C_12_ nanocage in polarizing its stable Si-C bond. Additionally, the energy gap of the pristine Si_12_C_12_ nanocage and Li/Li^+^ adsorbed Si_12_C_12_ nanocages were higher than the Ge_12_C_12_ nanocages, confirming the high stability of the Si-C bond compared to the Ge-C bond.

Furthermore, the chemical reactivity and kinetic stability of the endohedral O and Se doped nanocages were carefully assessed. The respective energy gap values of the endohedral doped nanocages without and with adsorption of Li atom and Li^+^ cation are presented in Table 4. As depicted in the table, it is fundamentally obvious that the O-Ge_12_C_12_ endohedral doped nanocage disclosed a lesser E_g_ value (1.85 eV) compared to the pristine Ge_12_C_12_ nanocage (2.01 eV). This result reflects that the highly electronegative O anion spreads its charge on the surface of the O-Ge_12_C_12_ endohedral doped nanocage, hence polarizing its Ge-C bond, which unveils it to chemical attack easily. Outstandingly, the energy gap of Li@O-Ge_12_C_12_ and Li^+^@O-Ge_12_C_12_ adsorbed nanocage showcased lower individual energy gaps of 1.77 eV (%∆E_g_= -4.32%) and 1.69 eV (%∆E_g_= -8.65%) in contrast to the O-Ge_12_C_12_ endohedral doped nanocage (1.85 eV) due to the combine charge transfer from Li/Li^+^ and O anion towards the surface of the nanocage, leading to softening of their Ge-C bonds, thus enhancing their reactivity towards a chemical reagent.

As for the O-Si_12_C_12_ endohedral doped nanocage, its energy gap value is slightly reduced compared to that of the pristine Si_12_C_12_ nanocage due to the effect of charge polarization of the Si-C bond. The energy gap reduction occurred with a slight variation, confirming the stability of the Si-C bond. This effect also reveals that the O-Si_12_C_12_ endohedral doped nanocage demonstrated a more significant energy gap (2.71 eV) than the O-Ge_12_C_12_ endohedral doped nanocage (1.85 eV). Astonishingly, Li@O-Si_12_C_12_ and Li^+^@O-Si_12_C_12_ adsorbed nanocages divulged lesser energy gap with respective values of 2.26 eV (%∆E_g_= -16.61%) and 2.62 eV (%∆E_g_= -3.32%) than O-Si_12_C_12_ endohedral doped nanocage, resulting from the influence of Si-C bond polarization by the joint charge transfer from the O anion and the adsorbed Li/Li^+^ towards the surface of their nanocages.

Transcending to the Se-Ge_12_C_12_ endohedral doped nanocage, it exhibited a lower energy gap (1.16 eV) in contrast to the pristine Ge_12_C_12_ nanocage (2.01 eV) and the O-Ge_12_C_12_ endohedral doped nanocage (1.85 eV). This phenomenal result is tied to the instability of the Se anion upon spreading its charge to the surface of Se-Ge_12_C_12_ endohedral doped nanocage, forming two Se-C bonds (Se_24_-C_3_ and Se_24_-C_4_) by sharing electrons, which destabilizes its cage as evident in Fig. 2. Remarkably, the formation of the two Se-C bonds is due to the similar electronegativity (2.55) of Se and C atoms in the Pauling’s electronegativity scale. Hence, the Se-C bonds are nonpolar covalent bonds^45^. Next, viewing the energy gaps of Li@Se-Ge_12_C_12_ and Li^+^@Se-Ge_12_C_12_ nanocages, it was observed that they augmented and stabilized their nanocages with specific energy gap values of 1.41 eV (%∆E_g_= 21.55%) and 1.35 eV (%∆E_g_= 16.35%) compared to the Se-Ge_12_C_12_ nanocage (1.16 eV). The stabilization of the Li@Se-Ge_12_C_12_ and Li^+^@Se-Ge_12_C_12_ nanocages can be related to charge transfer from the Li atom and Li^+^ cation towards the surface of the nanocage, countering the Se anion from sharing electrons with C atoms of the nanocages.

Systematically, Se-Si_12_C_12_ endohedral doped nanocage manifested a lower energy gap (1.85 eV) compared to the Si_12_C_12_ pristine nanocage (2.72 eV) and O-Si_12_C_12_ endohedral doped nanocage (2.71 eV). This impressive reduction in Se-Si_12_C_12_ endohedral doped nanocage is the combined effect of charge transfer from the Se anion to the nanocage surface and the increased atomic size of the Se anion, which tends to expand the nanocage, thereby elongating the stable Si-C bond. Compared to the effect of the bulkiness of Se and O endohedral doped Si_12_C_12_ nanocage, the Se anion has an average atomic radius of 120 pm^46^, while the O anion has an atomic radius of 73 pm^47^. Hence, atomic size was explicit in their chemical reactivity and kinetic stability. Further decrease in energy gap of Li@Se-Si_12_C_12_ and Li^+^@Se-Si_12_C_12_ nanocages with respective values of 1.75 eV (%∆E_g_= -5.41%) and 1.67 eV (%∆E_g_= -9.73%) compared to Se-Si_12_C_12_ endohedral doped nanocage (1.875 eV) is a result of triple effect of charge transfer from Se and adsorbed Li/Li^+^ to the surface of the nanocages and the bulkiness of the Se anion, constituting more polarizability and elongation of the Si-C bond, making it susceptible to chemical attack.

The density of states of the studied nanocages was also evaluated using the optimized geometry conducted at the DFT/B3LYP-GD3(BJ)/6-311 + G(d, p)/GEN/LanL2DZ level of theory with the aid of GaussSum software. Essentially, the density of states provides crucial information about the electrical conductivity of a molecular system concerning the energy gap. In this case, the electrical conductivity of a chemical system varies inversely with its energy gap. The relationship between the energy gap and the electrical conductivity of a molecular system is pointed out in Eq. (1).

This current study meticulously assessed the electrical conductivity of the investigated pristine, endohedral doped, and Li/Li^+^ adsorbed nanocages. The observed order of electrical conductivity of pristine and endohedral doped nanocages without Li/Li^+^ adsorption are as follows: Si_12_C_12_ < O-Si_12_C_12_ < Ge_12_C_12_ < O-Ge_12_C_12_ = Se-Si_12_C_12_ < Se-Ge_12_C_12_ with respective energy gap values of 2.72 > 2.71 > 2.01 > 1.85 = 1.85 > 1.16 eV.

Moreover, for the Li atom adsorbed pristine and endohedral doped nanocages, the trend of their electrical conductivity is laid out as follows: Li@Si_12_C_12_ < Li@O-Si_12_C_12_ < Li@O-Ge_12_C_12_ < Li@Se-Si_12_C_12_ < Li@Ge_12_C_12_ < Li@Se-Si_12_C_12_ with individual energy gap values of 2.69 > 2.26 > 1.77 > 1.75 > 1.68 > 1.41 eV.

**Fig. 5:**
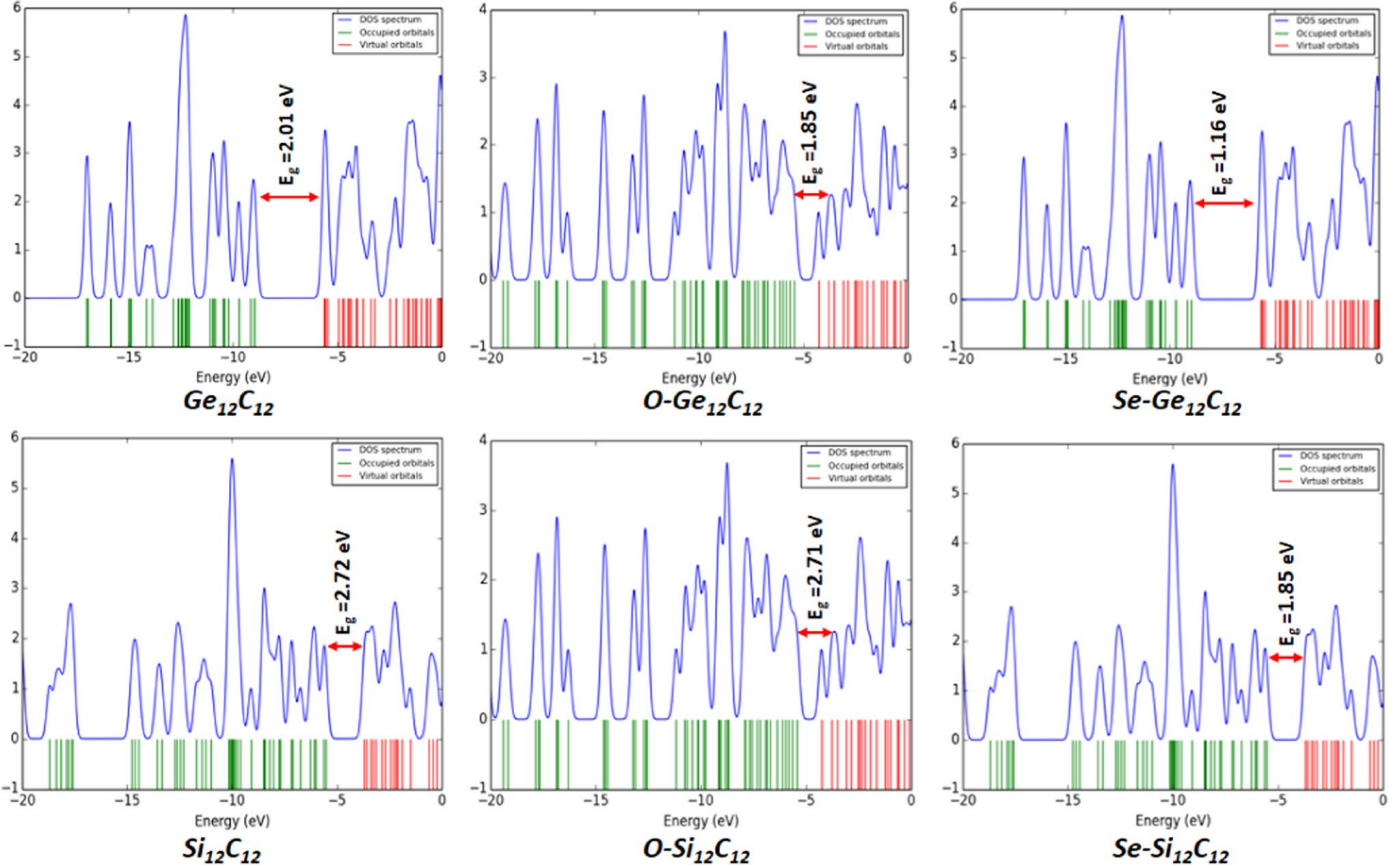
DOS plot of the pristine and endohedral doped nanocages without Li/Li^+^ adsorption.

**Fig. 6:**
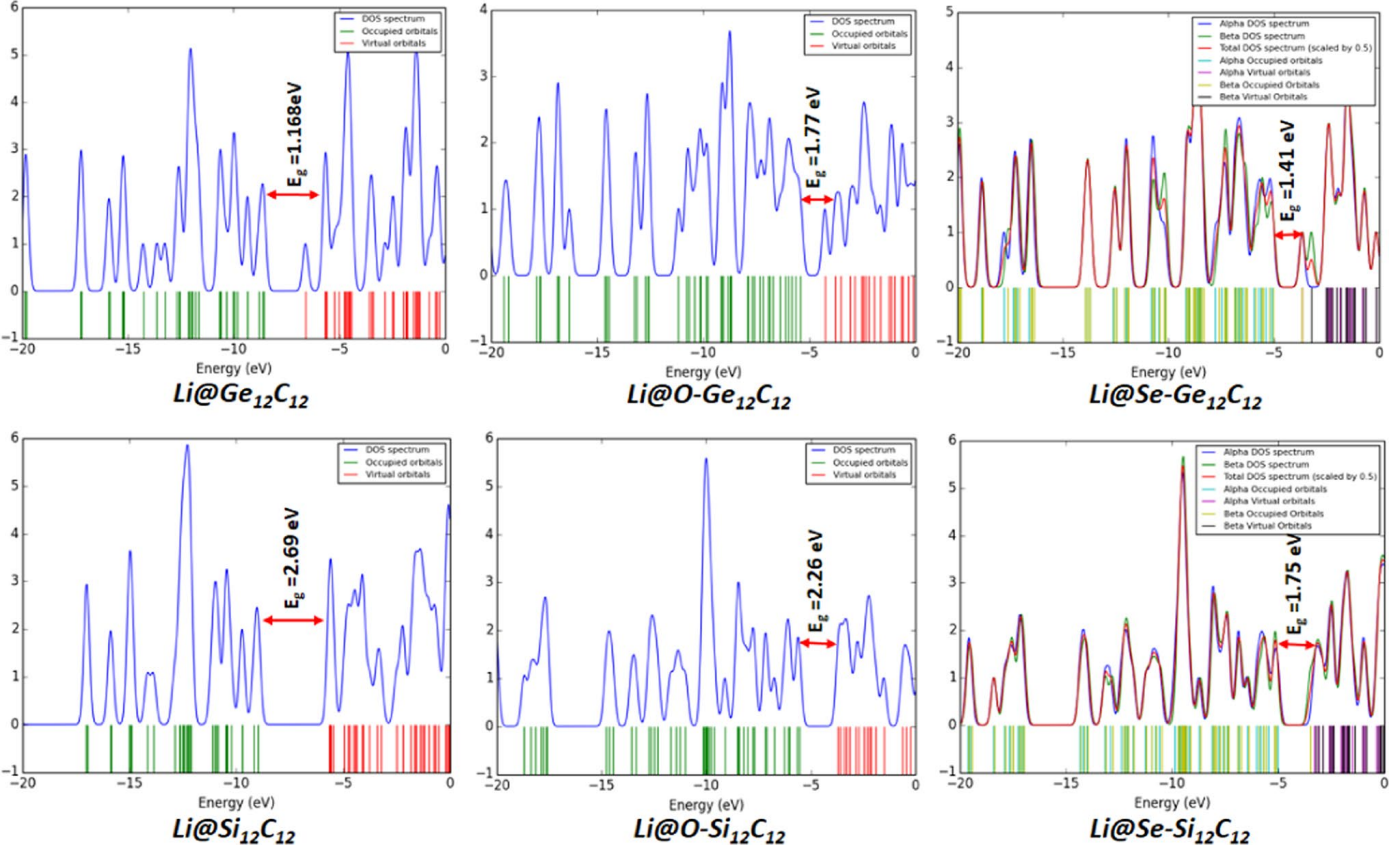
DOS plot of the pristine and endohedral doped nanocages with Li adsorption.

**Fig. 7:**
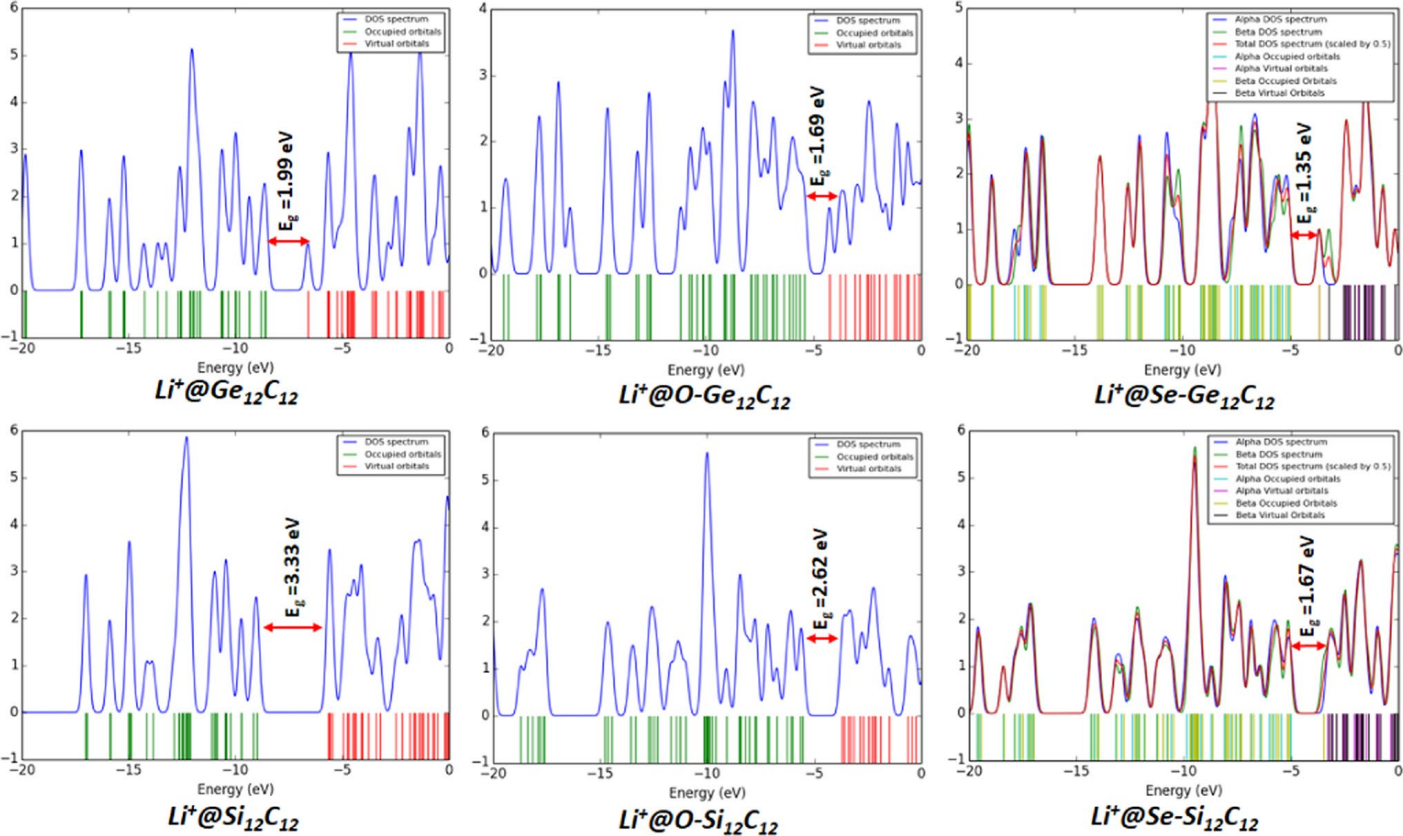
DOS plot of pristine and endohedral doped nanocages with Li^+^ adsorption.

To this end, the trend of electrical conductivity of Li^+^ cation adsorbed pristine and endohedral doped nanocages are showcased as follows: Li^+^@Si_12_C_12_ < Li^+^@O-Si_12_C_12_ < Li^+^@Ge_12_C_12_ < Li^+^@O-Ge_12_C_12_ < Li^+^@Se-Si_12_C_12_ < Li^+^@Se-Si_12_C_12_ with specific energy gap values of 3.33 > 2.62 > 1.99 > 1.69 > 1.67 > 1.35 eV. The DOS plot depicting the energy gaps for the electrical conductivity of the examined nanocages is displayed in Figs. 5 and 6, and 7.

**Table 3:** Electronic and electrochemical properties of pristine Ge_12_C_12_and Si_12_C_12_nanocages; HOMO energy (E_HOMO_), LUMO energy (E_LUMO_), energy gap (E_g_), change in electrochemical cell in Li/Li^+^adsorption (∆E_cell_), % Change in E_g_After Adsorption (%∆E_g_), Cell Voltage (V_cell_), Theoretical Capacity (C_th_) of the nanocages in Li-ion battery.

Structure	E_HOMO_ (eV)	E_LUMO_ (eV)	E_g_ (eV)	%∆E_g_	∆E_cell_ (kcal/mol)	V_cell_ (V)	C_th_ (mAh g^− 1^)
Ge_12_C_12_	-5.78	-3.77	2.01	–	-1.39	0.06	316.64
Li@Ge_12_C_12_	-4.37	-2.69	1.68	-16.42	–	–	–
Li^+^@Ge_12_C_12_	-8.59	-6.60	1.99	-1.00	–	–	–
Si_12_C_12_	-5.93	-3.21	2.72	–	-34.36	1.49	668.42
Li@Si_12_C_12_	-4.97	-2.28	2.69	-1.10	–	–	–
Li^+^@Si_12_C_12_	-8.97	-5.64	3.33	22.43	–	–	–

**Table 4:** Electronic and Electrochemical Properties of Endohedral Doped (O and Se) Ge_12_C_12_and Si_12_C_12_Nanocages; HOMO energy (E_HOMO_), LUMO energy (E_LUMO_), Energy gap (E_g_), Change in Electrochemical Cell in Li/Li^+^ Adsorption (∆E_cell_), % Change in E_g_After Adsorption (%∆E_g_), Cell Voltage (V_cell_), Theoretical Capacity (C_th_) of the nanocages in Li-ion battery.

Structure	E_HOMO_ (eV)	E_LUMO_ (eV)	E_g_ (eV)	%∆E_g_	∆E_cell_ (kcal/mol)	V_cell_ (V)	C_th_ (mAh g^− 1^)
O-Ge_12_C_12_	-5.77	-3.92	1.85	–	6.22	-0.27	266.31
Li@O-Ge_12_C_12_	-5.10	-3.33	1.77	-4.32	–	–	–
Li^+^@O-Ge_12_C_12_	-8.37	-6.68	1.69	-8.65	–	–	–
O-Si_12_C_12_	-5.93	-3.22	2.71	-	-3.23	0.14	477.78
Li@O-Si_12_C_12_	-4.99	-2.73	2.26	-16.61	–	–	–
Li^+^@O-Si_12_C_12_	-8.65	-6.03	2.62	-3.32	–	–	–
Se-Ge_12_C_12_	-5.42	-4.26	1.16	–	-2.54	0.11	163.81
Li@Se-Ge_12_C_12_	-5.08	-3.67	1.41	21.55	–	–	–
Li^+^@Se-Ge_12_C_12_	-8.35	-7.00	1.35	16.38	–	–	–
Se-Si_12_C_12_	-5.55	-3.70	1.85	–	-2.76	0.12	225.09
Li@Se-Si_12_C_12_	-5.01	-3.28	1.75	-5.41	–	–	–
Li^+^@Se-Si_12_C_12_	-8.28	-6.61	1.67	-9.73	–	–	–

### Adsorption of Li/Li^+^ on pristine Ge_12_C_12_ and Si_12_C_12_ nanocages

Adsorption of Li atom and Li^+^ cation on the C atom of pristine Ge_12_C_12_ nanocage were notable on bond Li_24_-C_3_ with respective bond lengths of 1.935 Å and 1.990 Å. Similarly, adsorption of the Li atom and Li^+^ cation on the C atom of pristine Si_12_C_12_ nanocage was observed on the Li_24_-C_7_ bond with individual bond distances of 1.973 Å and 1.996 Å. As laid out in Table 1., it is noteworthy that the bond distance of adsorption for Li^+^ cation on the C atom of the pristine Ge_12_C_12_ and Si_12_C_12_ nanocage was higher than the Li atom as a result of greater charge transfer, which enhances the polarizability of the Li-C bond.

The adsorption energy (E_ads_) of Li atom and Li^+^ cation on pristine Ge_12_C_12_ nanocage were computed to be -16.60 kcal/mol and − 17.99 kcal/mol, respectively. In comparison, the E_ads_ of Li atom and Li^+^ cation on the pristine Si_12_C_12_ nanocage corresponded to -37.36 kcal/mol and − 71.72 kcal/mol. Interestingly, Li^+^ cation adsorption on the pristine Ge_12_C_12_ and Si_12_C_12_ nanocages was stronger than the Li atom because the nanocages are more reactive towards the Li^+^ cation than the Li atom. Additionally, the weak interaction of the Li atom with pristine Ge_12_C_12_ and Si_12_C_12_ nanocages is linked to the presence of unpaired electrons in the valence shell of the Li atom^48^.

We also assessed the influence of Li atom and Li^+^ cation on the HOMO and LUMO orbitals of the pristine Ge_12_C_12_ and Si_12_C_12_ nanocages. In the case of adsorption of Li^+^ cation on the pristine Ge_12_C_12_ nanocage, the HOMO and LUMO were significantly stabilized from − 5.78 to -8.59 eV and − 3.77 to -6.60 eV, respectively, leading to considerable change in its energy gap. However, the adsorption of the Li atom on the pristine Ge_12_C_12_ nanocage drastically destabilized the HOMO and LUMO from − 5.78 to -4.37 eV and − 3.77 to -2.69 eV, correspondingly. The destabilization of the HOMO and LUMO upon adsorption of the Li atom is attributed to the presence of SOMO in its HOMO orbital, resulting in a drastic change in its E_g_ as seen in Table 3. In the same manner, Li^+^ cation adsorption on pristine Si_12_C_12_ nanocage stabilizes the HOMO and LUMO from − 5.93 to -8.97 eV and − 3.21 to -5.64 eV, respectively. In contrast, adsorption of the Li atom tremendously destabilized the HOMO and LUMO of pristine Si_12_C_12_ nanocage due to the visibility of SOMO in its HOMO orbital contributed from the unpaired electron in the Li atom. The destabilization of the HOMO and LUMO was observed to be -5.93 to -4.97 eV and − 3.21 to -2.28 eV, respectively.

### Adsorption of Li/Li^+^ on O-Ge_12_C_12_ and O-Si_12_C_12_ endohedral doped nanocages

The adsorption of Li atom and Li^+^ cation on the C atoms of O-Ge_12_C_12_ and O-Si_12_C_12_ endohedral doped nanocages was scientifically investigated. Here, the bond length of adsorption of the Li atom and Li^+^ cation on the C atom of O-Ge_12_C_12_ endohedral doped nanocage were on Li_25_-C_7_ bond, possessing individual bond distances of 1.958 Å and 2.004 Å. In essence, Li atom and Li^+^ cation adsorption on the O-Si_12_C_12_ endohedral doped nanocage was revealed on bond Li_25_-C_10_, with corresponding bond lengths of 1.957 Å and 2.003 Å. Again, the bond length of adsorption of Li^+^ cation on the O-Ge_12_C_12_ and O-Si_12_C_12_ endohedral doped nanocages were higher than Li atom adsorption, confirming greater polarization of their bond due to maximum charge transfer to the nanocages. Comparably, the bond length of adsorption for O-Ge_12_C_12_ and O-Si_12_C_12_ endohedral doped nanocages was observed to be higher than the pristine Ge_12_C_12_ and Si_12_C_12_ nanocages as a result of increased charge transfer from the endohedral doped O anion towards the surface of the nanocage, resulting in more polarization of their bond length of adsorption.

Concerning O-Ge_12_C_12_ endohedral doped nanocage, the estimated E_ads_ for Li atom and Li^+^ cation are − 62.03 kcal/mol and − 55.81 kcal/mol, correspondingly. Notably, E_ads_ for the Li atom are higher than Li^+^ cation, making O-Ge12C12 endohedral doped nanocages not a proper candidate for negative electrode material. As reported by several authors^49,50^, the E_ads_ of the Li atom must be lesser than the Li^+^ cation to portray a reasonable V_cell_ as anode material in rechargeable batteries. As for the adsorption of Li atom and Li^+^ cation on O-Si_12_C_12_ endohedral doped nanocage, the calculated E_ads_ are − 53.50 kcal/mol and − 56.73 kcal/mol, respectively.

Analyzing the effect of adsorption of Li atom and Li^+^ cation on the HOMO and LUMO of the O-Ge_12_C_12_ endohedral doped nanocage, it is obvious that Li^+^ cation stabilizes the HOMO and LUMO from − 5.77 to -8.37 eV and from − 3.92 to -6.68 eV, respectively. On the contrary, adsorption of Li atom on the O-Ge_12_O_12_ endohedral doped nanocage sharply destabilized the HOMO and LUMO from − 5.77 to -5.10 eV and − 3.92 to -3.33 eV, respectively. The destabilization effect on the HOMO and LUMO upon adsorption of the Li atom is also attributed to the presence of unpaired electron on the Li atom, leading to the formation of SOMO in the HOMO orbital of the O-Ge_12_C_12_ endohedral doped nanocage. Furthermore, we also checkmated the influence of adsorption of Li atom and Li^+^ cation on O-Si_12_C_12_ endohedral doped nanocage. Adsorption of Li^+^ cation stabilized the HOMO and LUMO of O-Si_12_C_12_ endohedral doped nanocage from − 5.93 to -8.65 eV and − 3.22 to -6.03 eV. Concerning Li atom adsorption on O-Si_12_C_12_ endohedral doped nanocage, the HOMO and LUMO were greatly destabilized from − 5.93 to -4.99 eV and − 3.22 to -2.73 eV, respectively. This destabilization of the HOMO and LUMO is chiefly linked to the excess electron in the s-orbital of the Li atom, contributing to the appearance of SOMO in the HOMO of the O-Si_12_C_12_ endohedral doped nanocage.

### Adsorption of Li/Li^+^ on Se-Ge_12_C_12_ and Se-Si_12_C_12_ endohedral doped nanocages

Li atom and Li^+^ cation adsorption on the C atoms of Se-Ge_12_C_12_ and Se-Si_12_C_12_ endohedral doped nanocages were meticulously scrutinized. The bond length of adsorption for Li atom and Li^+^ cation on Se-Ge_12_C_12_ endohedral doped nanocages were visible on the Li_24_-C_3_ bond with specific bond distances of 1.945 Å and 1.983 Å. For Li atom and Li^+^ cation adsorption on Se-Si_12_C_12_ endohedral doped nanocage, the bond length of adsorption was observed on the Li_25_-C_2_ bond with bond distances of 1.980 Å and 2.005 Å, respectively. In Se-Ge_12_C_12_ and Se-Si_12_C_12_ endohedral doped nanocages, the bond length of adsorption of Li^+^ cation is higher than Li atom adsorption, indicating larger charge transfer, which tends to polarize its bond.

The computed E_ads_ of Li atom and Li^+^ cation upon adsorption on Se-Ge_12_C_12_ endohedral doped nanocages were noted to be -19.60 kcal/mol and − 22.14 kcal/mol, respectively. Importantly, the E_ads_ of Li^+^ cation on Se-Ge_12_C_12_ endohedral doped nanocage slightly increased compared to pristine Ge_12_C_12_ nanocage and, hence, will experience a slight increase in its V_cell_ as anode material in LiBs. Crucially, the E_ads_ of Li atom and Li^+^ cation on Se-Si_12_C_12_ endohedral doped nanocage were estimated to be -55.81 kcal/mol and − 58.57 kcal/mol, respectively.

Wrapping it up here, we also investigated the effect of the adsorption of Li atom and Li^+^ on the HOMOs and LUMOs of Se-Ge_12_C_12_ and Se-Si_12_C_12_ endohedral doped nanocages. For the adsorption of Li^+^ cation on Se-Ge_12_C_12_ endohedral doped nanocages, the HOMO and LUMO were stabilized remarkably from − 5.42 to -8.35 eV and − 4.26 to -7.00 eV, correspondingly. In addition, adsorption of Li atom on Se-Ge_12_C_12_ endohedral doped nanocages destabilizes the HOMO and LUMO from − 5.42 to -5.08 eV and − 4.26 to -3.67 eV, respectively. Moreover, adsorption of Li^+^ cation on Se-Ge_12_C_12_ endohedral doped nanocage equally stabilized the HOMO and LUMO orbitals from − 5.55 to -8.28 eV and − 3.70 to -6.61 eV, respectively. For Li atom adsorption on Se-Ge_12_C_12_ endohedral doped nanocage, the HOMO and LUMO were destabilized from − 5.55 to -5.01 eV and − 3.70 to -3.28 eV, respectively. At this junction, we can infer that the destabilization of HOMO and LUMO upon adsorption of Li atom on Se-Ge_12_C_12_ endohedral doped nanocage is also likened to the presence of unpaired electron in the Li atom, forming a SOMO in the HOMO of the Li atom adsorbed Se-Ge_12_C_12_ endohedral doped nanocage.

### Application of pristine and endohedral doped (O and Se) Ge_12_C_12_and Si_12_C_12_nanocages as anode in LiBs

The electrochemical reactions persisting at the anode and cathode upon utilization of the studied pristine and endohedral doped nanocages as negative electrode material in lithium-ion batteries are reflected below.

11 $${\text{Anode}}{:}\,{\text{ Li}}@{\text{nanocage}}\leftrightarrow {\text{Li}}^{ + } @{\text{nanocage }} + {\text{ e}}^{{-}}$$

12 $${\text{Cathode}}:{\text{ Li}}^{ + } + {\text{ e}}^{{-}} \leftrightarrow {\text{Li}}$$

The overall cell reaction manifesting when the titled nanocages are applied in LiBs is depicted as follows.

13 $${\text{Li}}^{ + } + {\text{Li}}@{\text{nanocage}}\leftrightarrow{\text{Li}}^{ + } @{\text{nanocage }} + {\text{ Li }} + \Delta {\text{G}}_{{{\text{cell}}}}$$

The theoretical capacity (C_th_) of the nanocages was evaluated using the following equation.

14 $${\text{C}}_{{{\text{th}}}} = {\text{ nF}}/{\text{M}}_{{{\text{nanocage}}}}$$

In Eq. (14), n represents the number of stored Li, F denotes the Faraday constant (26801 mAh mol^− 1^), and M_nanocage_ designates the molar mass of one formula unit of the nanocages.

The cell voltage (V_cell_) of the scrutinized nanocages was computed by invoking the Nernst equation as seen below.

15 $${\text{V}}_{{{\text{cell}}}} = {\text{ }}{-}\Delta {\text{G}}_{{{\text{cell}}}} /{\text{zF}}$$

As postulated in Eq. (15), z signifies the charge of Li^+^ (cation in the electrolyte, z = 1), F demonstrates the Faraday’s constant (96,500 C/mol), and ∆G_cell_ symbolizes the Gibbs free energy change of the overall cell reaction. The DFT calculation of Gibbs free energy change was estimated at 0 K, and it is represented as follows:

16 $$\Delta {\text{G}}_{{{\text{cell}}}} = {\text{ }}\Delta {\text{E}}_{{{\text{cell}}}} + {\text{ P}}\Delta {\text{V }} + {\text{ T}}\Delta {\text{S}}$$

Previous reports have insinuated that the impact of volume and entropy are very minimal (< 0.01 V) on the V_cell_^11^. Hence, the V_cell_ of the Li^+^@nanocage or Li@nanocage was predicted from their internal energies as shown below.

17 $$\Delta {\text{G}}_{{{\text{cell}}}} \approx \Delta {\text{E}}_{{{\text{cell}}}} = {\text{ E}}\left( {{\text{Li}}^{ + } @{\text{nanocage}}} \right){\text{ }} + {\text{ E}}\left( {{\text{Li}}} \right){\text{ }}{-}{\text{ E}}({\text{Li}}@{\text{nanocage}}){-}{\text{ E}}\left( {{\text{Li}}^{ + } } \right)$$

Unambiguously, ∆E_cell_ is the difference between the adsorption energy of Li^+^ cation and Li atom interaction on the candidate nanocages^51^. From this line of reasoning, to obtain a reasonable ∆E_cell_, which corresponds to a better V_cell_, the adsorption of Li^+^ cation on the nanocages should be stronger with higher adsorption energy, while the adsorption of Li atom on the nanocages must be weaker with lesser adsorption energy than the Li^+^ cation. As positioned in Table 3, the ∆E_cell_, V_cell_, and C_th_ of the pristine Ge_12_C_12_ nanocage as anode material in LiBs were obtained as -1.39 kcal/mol, 0.06 V, and 316.64 mAh g^− 1^, respectively. As for pristine Si_12_C_12_ nanocage, the estimated ∆E_cell_, V_cell_, and C_th_ values as a negative electrode material in LiBs were − 34.36 kcal/mol, 1.49 V, and 668.42 mAh g^− 1^, correspondingly. Here, ∆E_cell_ and V_cell_ of pristine Si_12_C_12_ nanocage were noted to be higher than the pristine Ge_12_C_12_ nanocage due to its higher adsorption energy for Li^+^ cation interaction, as shown in Table 1. The obtained V_cell_ of pristine Si_12_C_12_ nanocage (1.49 V) is comparable to several nanostructured materials utilized in LiBs. For instance, Abedi et al. interacted Li on the N and P atoms of B_18_N_18_ and B_18_P_18_, respectively, possessing individual V_cell_ of 1.13 V and 1.31 V^52^. Hashemizadeh et al. also obtained V_cell_ of 1.03 V for C_32_ and 1.18 V for B_16_N_16_ when utilized in LiBs^17^. Additionally, Gao et al. achieved a V_cell_ of 1.45 V by applying (10,10) armchair carbon nanotubes as negative electrode material in LiBs^53^. Likewise, Chen et al. employed C_24_, Si_24_, B_21_N_21_, and Al_21_P_21_ as anode material in LiBs with individual V_cell_ of 0.88, 1.01, 1.24, and 1.43 V^18^. The theoretical capacity of the pristine Si_12_C_12_ nanocage (668.42 mAh g-1) is noteworthy as it compares favorably to values specified in the literature. To illustrate, Rehram et al. achieved a theoretical storage capacity of 921 mAh g^− 1^ using a Si_2_H_2_ sheet as anode material in LiBs^54^. In another research, Rehram et al. reported a maximum capacity of 410 mAh g^− 1^ by employing a 2D SnC sheet in LiBs^55^. Additionally, the computed theoretical capacity of the pristine Si_12_C_12_ nanocage is also comparable to the state-of-the-art graphite (372 mAh g^− 1^) and TiO_2_ (335 mAh g^− 1^) anodes utilized in LiBs^56,57^.

Principally, ∆E_cell_, V_cell,_ and C_th_ of the O-Ge_12_C_12_, O-Si_12_C_12_, Se-Ge_12_C_12_, and Se-Si_12_C_12_ endohedral doped nanocages are tabulated in Table 4. Based on the data from the table, it is evident that the O-Ge_12_C_12_ endohedral doped nanocage showed a positive ∆E_cell_ value of 6.23 kcal/mol and a negative V_cell_ value of -0.27 V. Therefore, it may not be suitable for practical use as a negative electrode material in LiBs. For the rest of the endohedral doped nanocages, the order of their electrochemical performance is as follows: O-Si_12_C_12_ > Se-Si_12_C_12_ > Se-Ge_12_C_12_ with specific ∆E_cell_ (V_cell_) C_th_ of -3.23 kcal/mol (0.14 V) 477.78 mAh g^− 1^, -2.76 kcal/mol (0.12 V) 225.09 mAh g^− 1^, and − 2.54 kcal/mol (0.11 V) 163.81 mAh g^− 1^. Overall, we can infer that pristine Si_12_C_12_ nanocage with the highest V_cell_ (1.49 V) and C_th_ (668.42 mAh g^− 1^) is the most suitable to be employed as anode material for lithium-ion batteries as it can demonstrate better discharge time and storage capacity compared to other simulated pristine and endohedral doped nanocages.

### Machine learning prediction of nanocages V_cell_

In this section, we used four regression algorithms (Linear, Lasso, Ridge, and ElasticNet regressions) to predict the V_cell_ of the studied nanocages. The input features for the prediction were the energy gap (Eg) and ∆E_cell_. To ensure consistency in predicting the V_cell_ of our studied nanocages using machine learning (ML) algorithms, we utilized the same hyperparameter tuning used for cross-validation in our regression models. Specifically, hyperparameters of regularized regression models were fine-tuned to achieve accurate prediction. The regularization hyperparameter (alpha) for Lasso regression was set at 0.001, while Ridge regression achieved optimal performance at alpha = 0.5. Similarly, ElasticNet regression performed better with alpha and l1_ratio hyperparameters set at 0.1.

Remarkably, our regression models from cross-validation disclosed R^2^ scores close to 1 (R^2^ = 0.99) with a slight deviation across each fold (R^2^ deviation < 0.001). This implies that the ML algorithms demonstrated excellent performance across each fold. Additionally, root mean square error (RMSE), which revealed how close our ML-predicted V_cells_ are to the DFT-predicted V_cells_, was observed to be less than 0.05, indicating an accurate prediction of our regression models. Linear regression model attained individual R^2^, R^2^ deviation, and RMSE of 0.99994, 0.00005, and 0.00390 across each fold. In contrast, Lasso regression demonstrated individual R^2^, R^2^ deviation, and RMSE values of 0.99999, 0.00000, and 0.00040. The absolute zero value of R^2^ deviation in Lasso regression depicts that the model performance generalized perfectly across the two k-folds. Furthermore, Ridge regression manifested R^2^, R^2^ deviation, and RMSE values of 0.99962, 0.00036, and 0.01024, respectively, while ElasticNet regression showcased specific R^2^, R^2^ deviation, and RMSE values of 0.99976, 0.00022, and 0.00812.

**Fig. 8:**
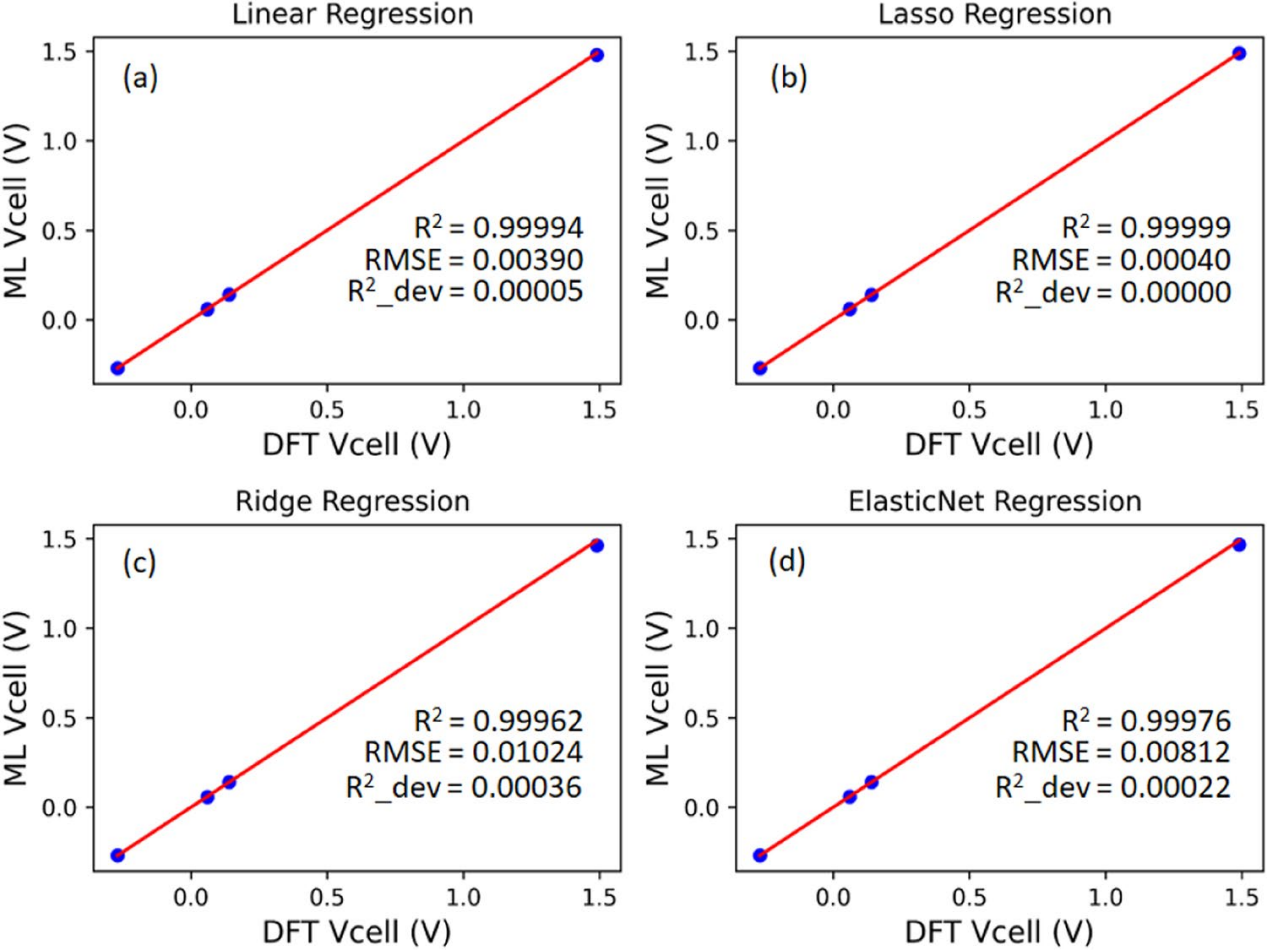
Plots of ML-predicted V_cell_ Vs DFT-predicted V_cell_ of nanocages using Y_train data (**a**) Linear regression (**b**) Lasso regression (**c**) Ridge regression (**d**) ElasticNet regression.

Generally, an excellent regression algorithm is characterized by higher R^2^ values with lower R^2^ deviation and RMSE. From this perspective, the order of our model’s generalization towards unseen data is as follows: Lasso regression > Linear regression > ElasticNet regression > Ridge regression. The exceptional performance of the Lasso regression model can be attributed to its L1 regularization technique that penalizes cost function by selecting essential features with their coefficients and discarding unimportant features with their coefficients. Hence, this ensures the robustness of the model in making accurate predictions. The plot of ML-predicted V_cell_ against DFT-predicted V_cell_ using split Y_train data is presented in Fig. 8.

## Conclusions

In this study, we conducted an in-depth assessment of the electronic and electrochemical performance of pristine and endohedral doped (O and Se) Ge_12_C_12_ and Si_12_C_12_ nanocages as anode material for LiBs using an advanced quantum mechanical approach at the DFT/B3LYP-GD3(BJ)/6-311 + G(d, p)/GEN/LanL2DZ level of theory. We also examined the electronic properties of the nanocages through frontier molecular orbital (FMO) analysis and density of states (DOS) and analyzed the adsorption energies of the Li/Li^+^ adsorbed nanocages to understand their storage capacity as negative electrode materials for LiBs.

Our key findings revealed that the pristine Si_12_C_12_ nanocage exhibited the highest stability among the studied nanocages without Li/Li^+^ adsorption, attributable to its stable Si-C bond. Additionally, endohedral doping of the studied nanocages enhanced their electronic properties, with the Se-Ge_12_C_12_ endohedral doped nanocage demonstrating the highest electrical conductivity due to its low energy gap value of 1.16 eV. We also observed that the interaction of Li/Li^+^ on the nanocages significantly influenced their electrical conductivity.

Furthermore, our analysis showed that the pristine Si_12_C_12_ nanocage displayed a significant V_cell_ and C_th_ of 1.49 V and 668.42 mAh g^− 1^, respectively, making it the most promising candidate for anode material in LiBs than counterpart nanocages. Moreover, we also utilized machine learning regression algorithms to predict the V_cell_ of the studied nanocages, achieving R^2^ scores close to 1 (R^2^ = 0.99) and lower RMSE values (RMSE < 0.05). Among the regression algorithms, Lasso regression demonstrated the best performance in predicting the V_cell_ of the nanocages, owing to its L1 regularization technique.

## Electronic supplementary material

Below is the link to the electronic supplementary material.

Supplementary Material 1

## Data Availability

All data are contained within the manuscript and supporting information. The machine learning code for the calculations presented in this work can be accessed via this GitHub repository:https://github.com/EgemonyeThankGod200/ThankGod_C_Egemonye_and_Tomsmith_O_Unimuke_Ge12C12_and_Si12C12_Nanostructure_Battery.
