# Supplementary material for: Machine learning-assisted DFT-prediction of pristine and endohedral doped (O and Se) Ge12C12 and Si12C12 nanostructures as anode materials for lithium-ion batteries
Source: Sci Rep. 2024 Oct 31;14:26244. doi: 10.1038/s41598-024-77150-x (PMC11528116; doi:10.1038/s41598-024-77150-x)
Supplement: Supplementary file 1 — Supplementary Material 1 [file 41598_2024_77150_MOESM1_ESM.docx]

**Supporting Information**

**Machine Learning-Assisted DFT-Prediction of Pristine and Endohedral Doped (O and Se) Ge_12_C_12_ and Si_12_C_12_ Nanostructures as Anode Materials for Lithium-ion Batteries**

**ThankGod C. Egemonye ^1*^ and Tomsmith O. Unimuke ^1*^**

^1^ Department of Pure and Applied Chemistry, University of Calabar, Calabar, Nigeria

***Corresponding author’s email:** [egemonyethankgod200@gmail.com](mailto:egemonyethankgod200@gmail.com), [tomsmithunimuke@unical.edu.ng](mailto:tomsmithunimuke@unical.edu.ng)

**Content**

- **SI section 1: Molecular Orbital Distribution**
- **SI section 2: Thermodynamics Properties of the Studied Nanostructures.**
- **SI section 3: XYZ Coordinates of Optimized Nanostructures.**
- **SI section 4: ML-Predicted V_cell_ Vs DFT-Predicted V_cell_ of the Studied Nanocages.**

**Molecular Orbital Distribution of the Studied Nanostructures.**

Molecular orbital distribution of the investigated nanocages within distinct energy levels was scrutinized. As visualized in **Fig. S1.** It can be seen that the HOMO of the pristine Ge_12_C_12_ nanocage is located on the C atoms of the nanocages, while the LUMO is evenly distributed on the C and Ge atoms. Likewise, the HOMO of the pristine Si_12_C_12_ nanocage was concentrated on the C atoms, while its LUMO was chiefly distributed on the C and Si atoms.

For the O-Ge_12_C_12_ endohedral doped nanocage, the highest occupied molecular orbitals (HOMOs) were greatly localized on the C atoms and O anion, while the lowest occupied molecular orbitals (LUMOs) were considerably spread on the C and Ge atoms. At this point, the presence of HOMO on the O anion of the O-Ge_12_C_12_ endohedral doped nanocage confirmed that charge was spread from the O anion towards the surface of the nanocage. Considering the HOMO-LUMO positions on the O-Si_12_C_12_ endohedral doped nanocage, the HOMOs were observed to be dominant on the O anion and C atoms, while the lowest occupied molecular orbitals LUMOs were equally spread on the Si and C atoms.

Similarly, the HOMO of the Se-Ge_12_C_12_ endohedral doped nanocage shifted heavily from the Se anion towards the C and Ge atoms as a result of the formation of Se-C bonds, while its LUMO is localized on Ge atoms. Symmetrically, the HOMO of the Se-Si_12_C_12_ endohedral doped nanocage is majorly concentrated on the Se anion and with minimal distribution on the C atoms, while the LUMO is widely spread on the Si and C atoms. The large shape of the HOMO on the Se anion indicates that maximum amount of charge was spread towards the surface of the nanocage, which is in tandem with the frontier molecular orbital analysis.

Uniformly, the adsorption of Li/Li^+^ on the pristine and endohedral doped nanocages has similar HOMO and LUMO distribution and, therefore, will be discussed concomitantly. The HOMO-LUMO plots of Li atom and Li^+^ cation adsorption on the studied nanocages are depicted in **Fig. S2 and S3.**, respectively. From Li@Ge_12_C_12_ and Li^+^@Ge_12_C_12_ nanocages, the HOMOs were distributed on the Ge and C atoms with greater concentration around those close to the point of Li/Li^+^ interaction, indicating great charge transfer towards them, while its LUMO is spread minimally around the C atoms. In Li@Si_12_C_12_ and Li^+^@Si_12_C_12_ nanocages, the HOMO is broadly concentrated around the adsorbed Li atom and Li cation, respectively. Meanwhile, the LUMO is predominantly located on the Ge and C atoms, with higher concentration observed for the Ge and C atoms near the adsorbed Li/Li^+^.

Remarkably, the HOMOs of Li@O-Ge_12_C_12_ and Li^+^@O-Ge_12_C_12_ endohedral doped nanocages were majorly spread on the endohedral doped O anion, Ge, and C atoms in proximity to the adsorbed Li/Li^+^, while the LUMOs were proportionally distributed on the Ge and C atoms. Systematically, the HOMOs of Li@O-Si_12_C_12_ and Li^+^@O-Si_12_C_12_ endohedral doped nanocages were predominant around the adsorbed Li atom and Li cation, with a minimal concentration on the C atoms close to the point of Li/Li^+^ adsorption on the nanocage, while its LUMO was positioned on the Si and C atoms.

Significantly, the HOMOs of Li@Se-Ge_12_C_12_ and Li^+^@Se-Ge_12_C_12_ endohedral doped nanocage were largely concentrated around the endohedral doped Se anion and appeared smaller around Li/Li^+^, depicting that greater charge was transferred from the Se anion towards the surface of the nanocage than from the Li atom and Li cation, while their LUMO is circulated on the Ge and C atoms. Furthermore, the HOMOs of Li@Se-Si_12_C_12_ and Li^+^@Se-Si_12_C_12_ endohedral doped nanocages were significantly pronounced around the Se anion with minimal effect on the C atoms, while the LUMO is visible on the Si and C atoms. More interestingly, the location of the HOMO on the Se anion also confirmed that a significant charge was spread to the cages, resulting in more polarization and destabilization of the Si-C bonds.


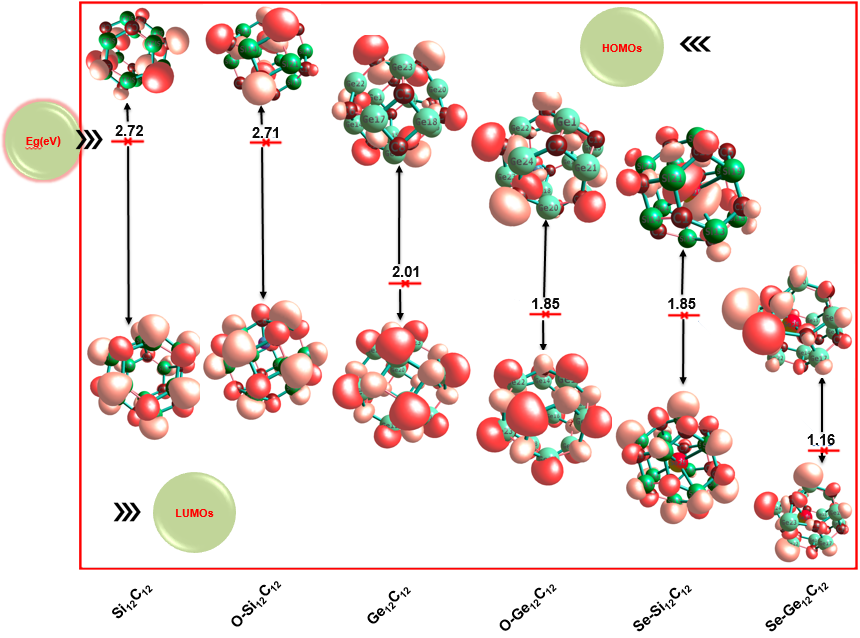


**Fig. S1.** HOMO-LUMO plot of pristine and endohedral doped nanocages without Li/Li^+^ adsorption.


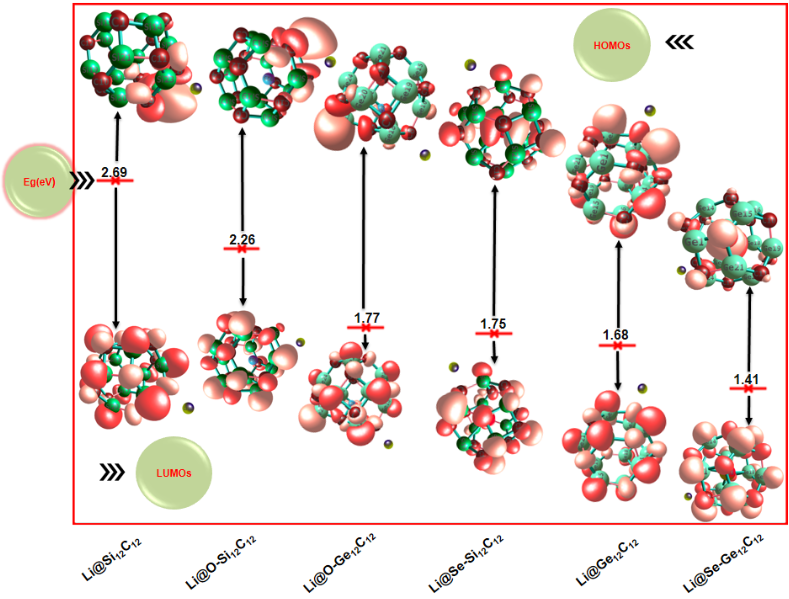


**Fig. S2.** HOMO-LUMO plot of pristine and endohedral doped nanocages with Li atom adsorption.


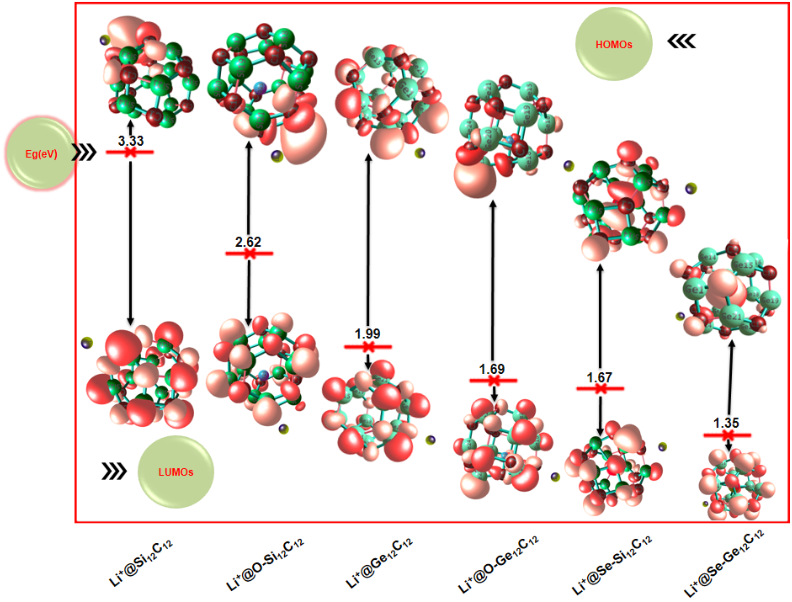


**Fig. S3.** HOMO-LUMO plot of pristine and endohedral doped nanocages with Li^+^ cation adsorption.

**Table S1. Thermodynamics Properties of Pristine and Endohedral Doped (O and Se) Ge_12_C_12_ and Si_12_C_12_ Nanocages; Standard Enthalpy of Formation (∆H_f_^⁰^) and Standard Gibbs Free Energy of Formation (∆G_f_^⁰^) of the Nanocages**

| **Nanocages** | **∆H_f_^⁰^ (kcal/mol)** | **∆G_f_^⁰^ (kcal/mol)** |
| --- | --- | --- |
|  |  |  |
| Ge_12_C_12_ | - | - |
| Li@Ge_12_C_12_ | -370.65 | -365.68 |
| Li^+^@Ge_12_C_12_ | -372.85 | -366.07 |
| Si_12_C_12_ | **-** | **-** |
| Li@Si_12_C_12_ | -45.77 | -38.80 |
| Li^+^@Si_12_C_12_ | -51.23 | -43.44 |
| O-Ge_12_C_12_ | **-** | **-** |
| Li@O-Ge_12_C_12_ | -60.98 | -54.79 |
| Li^+^@O-Ge_12_C_12_ | -55.11 | -57.02 |
| O-Si_12_C_12_ | **-** | **-** |
| Li@O-Si_12_C_12_ | -52.58 | -46.04 |
| Li^+^@O-Si_12_C_12_ | -56.08 | -48.81 |
| Se-Ge_12_C_12_ | **-** | **-** |
| Li@Se-Ge_12_C_12_ | -245.31 | -235.69 |
| Li^+^@Se-Ge_12_C_12_ | -248.05 | -237.03 |
| Se-Si_12_C_12_ | **-** | **-** |
| Li@Se-Si_12_C_12_ | -55.21 | -48.14 |
| Li^+^@Se-Si_12_C_12_ | -57.97 | -49.37 |

**Table S1.** represents the standard enthalpy of formation and standard Gibbs free energy of formation of the investigated nanostructures. Lower values signify a more thermodynamically stable nanostructure.

**Table S2. Cartesian Coordinates of Pristine Ge_12_C_12_** **Nanocage.**

| **Center**  **Number** | **Atomic**  **Number** | **Atomic**  **Type** | **Coordinates (Angstroms)** | | |
| --- | --- | --- | --- | --- | --- |
|  |  |  | **X** | **Y** | **Z** |
| 1 | 32 | 0 | 3.055913 | 0.011470 | 1.213159 |
| 2 | 6 | 0 | -2.328428 | 1.429457 | -0.002144 |
| 3 | 6 | 0 | -1.429326 | -0.011343 | 2.327957 |
| 4 | 6 | 0 | -0.006583 | 2.328346 | 1.429363 |
| 5 | 6 | 0 | 2.328414 | 1.429480 | 0.002139 |
| 6 | 6 | 0 | 1.429333 | 0.011351 | 2.327953 |
| 7 | 6 | 0 | 1.429326 | -0.011337 | -2.327957 |
| 8 | 6 | 0 | 2.328428 | -1.429457 | -0.002146 |
| 9 | 6 | 0 | 0.006588 | -2.328341 | 1.429370 |
| 10 | 6 | 0 | -0.006565 | -2.328346 | -1.429363 |
| 11 | 6 | 0 | -1.429333 | 0.011329 | -2.327953 |
| 12 | 6 | 0 | -2.328414 | -1.429480 | 0.002151 |
| 13 | 6 | 0 | 0.006560 | 2.328341 | -1.429370 |
| 14 | 32 | 0 | 0.020830 | -1.213035 | 3.055551 |
| 15 | 32 | 0 | -1.213038 | -3.055775 | 0.020384 |
| 16 | 32 | 0 | 1.213068 | -3.055763 | -0.020374 |
| 17 | 32 | 0 | 3.055916 | -0.011466 | 1.213150 |
| 18 | 32 | 0 | 3.055912 | 0.011497 | -1.213159 |
| 19 | 32 | 0 | -0.020818 | -1.213045 | -3.055547 |
| 20 | 32 | 0 | 0.020809 | 1.213035 | -3.055551 |
| 21 | 32 | 0 | -3.055916 | -0.011501 | -1.213150 |
| 22 | 32 | 0 | -0.020821 | 1.213045 | 3.055547 |
| 23 | 32 | 0 | 1.213038 | 3.055775 | 0.020370 |
| 24 | 32 | 0 | -1.213068 | 3.055763 | -0.020380 |

**Table S3. Cartesian Coordinates of Pristine Si_12_C_12_** **Nanocage.**

| **Center**  **Number** | **Atomic**  **Number** | **Atomic**  **Type** | **Coordinates (Angstroms)** | | |
| --- | --- | --- | --- | --- | --- |
|  |  |  | **X** | **Y** | **Z** |
| 1 | 6 | 0 | 2.258337 | 1.393869 | 0.002074 |
| 2 | 6 | 0 | 1.393730 | -0.010508 | -2.257889 |
| 3 | 6 | 0 | 0.006025 | 2.258251 | -1.393781 |
| 4 | 6 | 0 | -2.258333 | 1.393874 | -0.002081 |
| 5 | 6 | 0 | -1.393757 | 0.010498 | -2.257872 |
| 6 | 6 | 0 | -1.393730 | -0.010494 | 2.257889 |
| 7 | 6 | 0 | -2.258337 | -1.393869 | 0.002108 |
| 8 | 6 | 0 | -0.006042 | -2.258258 | -1.393770 |
| 9 | 6 | 0 | 0.006036 | -2.258251 | 1.393781 |
| 10 | 6 | 0 | 1.393757 | 0.010505 | 2.257872 |
| 11 | 6 | 0 | 2.258333 | -1.393874 | -0.002101 |
| 12 | 6 | 0 | -0.006020 | 2.258258 | 1.393770 |
| 13 | 14 | 0 | 1.167055 | 2.975016 | 0.019917 |
| 14 | 14 | 0 | 2.975163 | 0.011513 | -1.167166 |
| 15 | 14 | 0 | 0.020049 | 1.167036 | -2.974809 |
| 16 | 14 | 0 | -2.975177 | -0.011518 | -1.167131 |
| 17 | 14 | 0 | -2.975163 | 0.011525 | 1.167166 |
| 18 | 14 | 0 | -1.167047 | 2.975019 | -0.019931 |
| 19 | 14 | 0 | -0.020047 | 1.167050 | 2.974804 |
| 20 | 14 | 0 | 0.020082 | -1.167036 | 2.974809 |
| 21 | 14 | 0 | 2.975177 | -0.011520 | 1.167131 |
| 22 | 14 | 0 | -1.167054 | -2.975016 | 0.019945 |
| 23 | 14 | 0 | 1.167047 | -2.975019 | -0.019931 |
| 24 | 14 | 0 | -0.020085 | -1.167050 | -2.974803 |

**Table S4. Cartesian Coordinates of O-Ge_12_C_12_ Endohedral Doped** **Nanocage**

| **Center**  **Number** | **Atomic**  **Number** | **Atomic**  **Type** | **Coordinates (Angstroms)** | | |
| --- | --- | --- | --- | --- | --- |
|  |  |  | **X** | **Y** | **Z** |
| 1 | 32 | 0 | 2.329049 | -0.975503 | 1.596392 |
| 2 | 6 | 0 | 3.247715 | 0.100844 | 0.194515 |
| 3 | 6 | 0 | 1.307398 | -2.659951 | 1.344030 |
| 4 | 6 | 0 | 1.493912 | -1.941235 | -2.142639 |
| 5 | 6 | 0 | -1.778560 | -0.622237 | -2.651931 |
| 6 | 6 | 0 | -1.204352 | -3.021292 | -0.078407 |
| 7 | 6 | 0 | -1.304997 | 2.659988 | -1.343901 |
| 8 | 6 | 0 | -3.245314 | -0.100807 | -0.194386 |
| 9 | 6 | 0 | -1.441216 | -0.897800 | 2.775166 |
| 10 | 6 | 0 | -1.491510 | 1.941272 | 2.142769 |
| 11 | 6 | 0 | 1.206754 | 3.021329 | 0.078537 |
| 12 | 6 | 0 | 1.780961 | 0.622274 | 2.652061 |
| 13 | 6 | 0 | 1.443617 | 0.897837 | -2.775037 |
| 14 | 32 | 0 | -0.627927 | -2.364954 | 1.711755 |
| 15 | 32 | 0 | -0.188261 | 0.644821 | 2.909895 |
| 16 | 32 | 0 | -2.501021 | 0.312106 | 1.600148 |
| 17 | 32 | 0 | -2.280337 | -1.638610 | -1.013965 |
| 18 | 32 | 0 | -2.326647 | 0.975540 | -1.596262 |
| 19 | 32 | 0 | -0.720224 | 2.845110 | 0.551224 |
| 20 | 32 | 0 | 0.630328 | 2.364990 | -1.711625 |
| 21 | 32 | 0 | 2.282739 | 1.638647 | 1.014095 |
| 22 | 32 | 0 | 0.722625 | -2.845074 | -0.551095 |
| 23 | 32 | 0 | 0.190662 | -0.644784 | -2.909765 |
| 24 | 32 | 0 | 2.503422 | -0.312070 | -1.600018 |
| 25 | 8 | 0 | -0.068439 | -0.001043 | -0.003699 |

**Table S5. Cartesian Coordinates of O-Si_12_C_12_** **Endohedral Doped Nanocage.**

| **Center**  **Number** | **Atomic**  **Number** | **Atomic**  **Type** | **Coordinates (Angstroms)** | | |
| --- | --- | --- | --- | --- | --- |
|  |  |  | **X** | **Y** | **Z** |
| 1 | 6 | 0 | 0.910057 | -1.984177 | -1.995880 |
| 2 | 6 | 0 | -2.099591 | -0.872477 | -1.889772 |
| 3 | 6 | 0 | 0.303765 | 1.079974 | -2.736489 |
| 4 | 6 | 0 | 0.604879 | 2.893372 | -0.104753 |
| 5 | 6 | 0 | -2.259369 | 1.681214 | -0.899653 |
| 6 | 6 | 0 | 2.102255 | 0.872502 | 1.890006 |
| 7 | 6 | 0 | -0.907393 | 1.984203 | 1.996114 |
| 8 | 6 | 0 | -2.584674 | -0.656539 | 1.276168 |
| 9 | 6 | 0 | -0.301101 | -1.079949 | 2.736723 |
| 10 | 6 | 0 | 2.262034 | -1.681188 | 0.899887 |
| 11 | 6 | 0 | -0.602215 | -2.893346 | 0.104987 |
| 12 | 6 | 0 | 2.587338 | 0.656565 | -1.275934 |
| 13 | 14 | 0 | 1.525441 | -0.408575 | -2.501271 |
| 14 | 14 | 0 | -0.987866 | -2.227057 | -1.675724 |
| 15 | 14 | 0 | -1.423344 | 0.858953 | -2.445147 |
| 16 | 14 | 0 | -1.293043 | 2.650492 | 0.215403 |
| 17 | 14 | 0 | 0.990530 | 2.227083 | 1.675958 |
| 18 | 14 | 0 | 1.365662 | 2.145115 | -1.511152 |
| 19 | 14 | 0 | 2.938280 | 0.050242 | 0.344513 |
| 20 | 14 | 0 | 1.426008 | -0.858928 | 2.445380 |
| 21 | 14 | 0 | 1.295707 | -2.650466 | -0.215169 |
| 22 | 14 | 0 | -1.522777 | 0.408601 | 2.501505 |
| 23 | 14 | 0 | -1.362998 | -2.145089 | 1.511386 |
| 24 | 14 | 0 | -2.935616 | -0.050216 | -0.344279 |
| 25 | 8 | 0 | -0.039961 | -0.000387 | -0.003505 |

**Table S6. Cartesian Coordinates of Se-Ge_12_C_12_** **Endohedral Doped Nanocage.**

| **Center**  **Number** | **Atomic**  **Number** | **Atomic**  **Type** | **Coordinates (Angstroms)** | | |
| --- | --- | --- | --- | --- | --- |
|  |  |  | **X** | **Y** | **Z** |
| 1 | 32 | 0 | 0.198181 | -2.547275 | 1.388582 |
| 2 | 6 | 0 | 2.087166 | -2.056632 | 1.127840 |
| 3 | 6 | 0 | -1.079428 | -3.002449 | 0.077536 |
| 4 | 6 | 0 | 1.464689 | -1.947552 | -2.271413 |
| 5 | 6 | 0 | -0.009966 | 1.261304 | -2.817471 |
| 6 | 6 | 0 | -2.214953 | -1.402764 | -2.037250 |
| 7 | 6 | 0 | 1.089057 | 3.015185 | -0.063173 |
| 8 | 6 | 0 | -2.187936 | 2.142854 | -1.157957 |
| 9 | 6 | 0 | -2.880842 | -0.314899 | 1.155521 |
| 10 | 6 | 0 | -1.294264 | 1.817735 | 2.119606 |
| 11 | 6 | 0 | 2.092968 | 1.293479 | 1.949775 |
| 12 | 6 | 0 | -0.021475 | -1.251971 | 2.811062 |
| 13 | 6 | 0 | 3.047201 | 0.307325 | -1.209195 |
| 14 | 32 | 0 | -2.522215 | -1.679461 | -0.105700 |
| 15 | 32 | 0 | -1.447644 | -0.083824 | 2.436523 |
| 16 | 32 | 0 | -2.482224 | 1.521627 | 0.607242 |
| 17 | 32 | 0 | -1.744763 | 0.475457 | -2.135524 |
| 18 | 32 | 0 | -0.262231 | 2.544573 | -1.310479 |
| 19 | 32 | 0 | 0.458749 | 2.305818 | 1.644250 |
| 20 | 32 | 0 | 2.482188 | 1.617419 | 0.078439 |
| 21 | 32 | 0 | 1.682528 | -0.514346 | 2.248810 |
| 22 | 32 | 0 | -0.405994 | -2.177096 | -1.563261 |
| 23 | 32 | 0 | 1.464934 | 0.083649 | -2.356381 |
| 24 | 32 | 0 | 2.538964 | -1.494175 | -0.601188 |
| 25 | 34 | 0 | 0.020928 | -0.024864 | -0.256215 |

**Table S7. Cartesian Coordinates of Se-Si_12_C_12_** **Endohedral Doped Nanocage.**

| **Center**  **Number** | **Atomic**  **Number** | **Atomic**  **Type** | **Coordinates (Angstroms)** | | |
| --- | --- | --- | --- | --- | --- |
|  |  |  | **X** | **Y** | **Z** |
| 1 | 6 | 0 | -2.779325 | -0.868843 | 0.429566 |
| 2 | 6 | 0 | -0.756661 | -2.404630 | -1.533927 |
| 3 | 6 | 0 | -0.258536 | -2.447672 | 1.637056 |
| 4 | 6 | 0 | 2.226927 | -0.435263 | 1.915981 |
| 5 | 6 | 0 | 1.864413 | -2.177624 | -0.755699 |
| 6 | 6 | 0 | 0.785934 | 2.408408 | 1.538898 |
| 7 | 6 | 0 | 2.808598 | 0.872621 | -0.424595 |
| 8 | 6 | 0 | 0.852457 | 0.050387 | -2.833444 |
| 9 | 6 | 0 | 0.287809 | 2.451450 | -1.632085 |
| 10 | 6 | 0 | -1.835140 | 2.181402 | 0.760670 |
| 11 | 6 | 0 | -2.197653 | 0.439041 | -1.911010 |
| 12 | 6 | 0 | -0.823184 | -0.046608 | 2.838416 |
| 13 | 14 | 0 | -1.851397 | -1.360643 | 1.848622 |
| 14 | 14 | 0 | -2.206165 | -1.415433 | -1.341402 |
| 15 | 14 | 0 | 0.263040 | -2.945069 | 0.025475 |
| 16 | 14 | 0 | 2.800087 | -0.981853 | 0.145013 |
| 17 | 14 | 0 | 2.235438 | 1.419211 | 1.346373 |
| 18 | 14 | 0 | 0.769677 | -1.133638 | 2.626850 |
| 19 | 14 | 0 | -0.815439 | 1.640963 | 2.320072 |
| 20 | 14 | 0 | -0.233767 | 2.948847 | -0.020504 |
| 21 | 14 | 0 | -2.770814 | 0.985631 | -0.140042 |
| 22 | 14 | 0 | 1.880670 | 1.364421 | -1.843651 |
| 23 | 14 | 0 | -0.740404 | 1.137416 | -2.621879 |
| 24 | 14 | 0 | 0.844712 | -1.637185 | -2.315101 |
| 25 | 34 | 0 | -0.103317 | -0.013335 | -0.017545 |

**Table S8. Cartesian Coordinates of Pristine Li@Ge_12_C_12_** **Nanocage.**

| **Center**  **Number** | **Atomic**  **Number** | **Atomic**  **Type** | **Coordinates (Angstroms)** | | |
| --- | --- | --- | --- | --- | --- |
|  |  |  | **X** | **Y** | **Z** |
| 1 | 32 | 0 | -0.638575 | -1.311975 | -2.519144 |
| 2 | 6 | 0 | -1.321202 | 0.486333 | -2.721450 |
| 3 | 6 | 0 | -1.203426 | -2.533077 | -1.238670 |
| 4 | 6 | 0 | -3.041204 | -0.060982 | 0.118469 |
| 5 | 6 | 0 | -1.114474 | 0.721666 | 2.764862 |
| 6 | 6 | 0 | -1.099876 | -2.412818 | 1.541044 |
| 7 | 6 | 0 | 1.277752 | 2.532500 | 1.238856 |
| 8 | 6 | 0 | 1.394173 | -0.485957 | 2.721639 |
| 9 | 6 | 0 | 1.909923 | -2.444902 | 0.034788 |
| 10 | 6 | 0 | 3.114011 | 0.061061 | -0.119425 |
| 11 | 6 | 0 | 1.171258 | 2.413666 | -1.540813 |
| 12 | 6 | 0 | 1.187211 | -0.721775 | -2.764624 |
| 13 | 6 | 0 | -1.837013 | 2.445076 | -0.033408 |
| 14 | 32 | 0 | 0.115564 | -2.916629 | 0.123444 |
| 15 | 32 | 0 | 2.326193 | -1.184908 | -1.372433 |
| 16 | 32 | 0 | 2.426850 | -1.068506 | 1.292024 |
| 17 | 32 | 0 | -0.442374 | -1.089833 | 2.666425 |
| 18 | 32 | 0 | 0.710758 | 1.312434 | 2.519368 |
| 19 | 32 | 0 | 2.361282 | 1.758609 | -0.164430 |
| 20 | 32 | 0 | -0.042624 | 2.916874 | -0.121006 |
| 21 | 32 | 0 | 0.515902 | 1.089644 | -2.666222 |
| 22 | 32 | 0 | -2.288518 | -1.758577 | 0.162410 |
| 23 | 32 | 0 | -2.254175 | 1.183282 | 1.372754 |
| 24 | 32 | 0 | -2.353145 | 1.070383 | -1.291918 |
| 25 | 3 | 0 | -5.537061 | -0.010094 | -0.016104 |

**Table S9. Cartesian Coordinates of Pristine Li@Si_12_C_12_** **Nanocage.**

| **Center**  **Number** | **Atomic**  **Number** | **Atomic**  **Type** | **Coordinates (Angstroms)** | | |
| --- | --- | --- | --- | --- | --- |
|  |  |  | **X** | **Y** | **Z** |
| 1 | 6 | 0 | -1.819667 | -1.479769 | -1.833350 |
| 2 | 6 | 0 | 0.680753 | -2.819452 | -0.352405 |
| 3 | 6 | 0 | -1.870786 | -1.890408 | 1.340552 |
| 4 | 6 | 0 | -0.711581 | 0.726322 | 2.771904 |
| 5 | 6 | 0 | 1.251955 | -1.684928 | 2.013715 |
| 6 | 6 | 0 | -0.812920 | 2.818872 | 0.353067 |
| 7 | 6 | 0 | 1.689209 | 1.479960 | 1.833269 |
| 8 | 6 | 0 | 2.800599 | -0.424668 | -0.486510 |
| 9 | 6 | 0 | 1.740583 | 1.890777 | -1.339784 |
| 10 | 6 | 0 | -1.380567 | 1.685485 | -2.014454 |
| 11 | 6 | 0 | 0.581285 | -0.726515 | -2.771868 |
| 12 | 6 | 0 | -2.931191 | 0.424284 | 0.485687 |
| 13 | 14 | 0 | -2.560218 | -1.232186 | -0.239692 |
| 14 | 14 | 0 | -0.095733 | -2.138715 | -1.795704 |
| 15 | 14 | 0 | -0.207944 | -2.501241 | 1.233816 |
| 16 | 14 | 0 | 0.960623 | -0.034379 | 2.597067 |
| 17 | 14 | 0 | -0.034145 | 2.138861 | 1.795477 |
| 18 | 14 | 0 | -2.026168 | -0.166211 | 1.981503 |
| 19 | 14 | 0 | -2.176460 | 1.794347 | -0.352877 |
| 20 | 14 | 0 | 0.078027 | 2.502186 | -1.231868 |
| 21 | 14 | 0 | -1.091491 | 0.034221 | -2.596902 |
| 22 | 14 | 0 | 2.430468 | 1.230734 | 0.240195 |
| 23 | 14 | 0 | 1.895130 | 0.167646 | -1.982070 |
| 24 | 14 | 0 | 2.045599 | -1.795313 | 0.350859 |
| 25 | 3 | 0 | 5.215441 | 0.000307 | 0.001277 |

**Table S10. Cartesian Coordinates of Li@O-Ge_12_C_12_ Endohedral Doped** **Nanocage.**

| **Center**  **Number** | **Atomic**  **Number** | **Atomic**  **Type** | **Coordinates (Angstroms)** | | |
| --- | --- | --- | --- | --- | --- |
|  |  |  | **X** | **Y** | **Z** |
| 1 | 32 | 0 | 1.908598 | 1.343358 | -1.803476 |
| 2 | 6 | 0 | 2.969094 | 0.006793 | -0.916410 |
| 3 | 6 | 0 | 1.028560 | 2.735681 | -0.944369 |
| 4 | 6 | 0 | 2.003795 | 1.421810 | 1.968237 |
| 5 | 6 | 0 | -0.909508 | -0.004877 | 2.625785 |
| 6 | 6 | 0 | -1.009751 | 2.901337 | 0.954336 |
| 7 | 6 | 0 | -0.998404 | -2.906807 | 0.947802 |
| 8 | 6 | 0 | -3.185363 | -0.006762 | 0.921817 |
| 9 | 6 | 0 | -1.854866 | 1.392525 | -1.942723 |
| 10 | 6 | 0 | -1.849917 | -1.395149 | -1.945782 |
| 11 | 6 | 0 | 1.039297 | -2.730149 | -0.950426 |
| 12 | 6 | 0 | 1.050997 | 0.004880 | -2.902763 |
| 13 | 6 | 0 | 2.008802 | -1.418408 | 1.964897 |
| 14 | 32 | 0 | -0.886422 | 2.649869 | -0.967048 |
| 15 | 32 | 0 | -0.790775 | 0.001651 | -2.780517 |
| 16 | 32 | 0 | -2.721389 | -0.003942 | -0.969917 |
| 17 | 32 | 0 | -1.891310 | 1.401303 | 1.644318 |
| 18 | 32 | 0 | -1.886046 | -1.412151 | 1.641328 |
| 19 | 32 | 0 | -0.875946 | -2.650273 | -0.972880 |
| 20 | 32 | 0 | 0.953385 | -2.599890 | 0.964409 |
| 21 | 32 | 0 | 1.913920 | -1.332181 | -1.806073 |
| 22 | 32 | 0 | 0.943186 | 2.600487 | 0.969994 |
| 23 | 32 | 0 | 1.029639 | -0.001042 | 2.786793 |
| 24 | 32 | 0 | 2.854419 | 0.004283 | 0.927150 |
| 25 | 8 | 0 | -0.501576 | -0.002193 | 1.097926 |
| 26 | 3 | 0 | -5.128041 | -0.011624 | 1.414540 |

**Table S11. Cartesian Coordinates of Li@O-Si_12_C_12_ Endohedral Doped** **Nanocage.**

| **Center**  **Number** | **Atomic**  **Number** | **Atomic**  **Type** | **Coordinates (Angstroms)** | | |
| --- | --- | --- | --- | --- | --- |
|  |  |  | **X** | **Y** | **Z** |
| 1 | 6 | 0 | 1.881881 | 1.564869 | 1.560931 |
| 2 | 6 | 0 | 1.163805 | 2.285859 | -1.460635 |
| 3 | 6 | 0 | -1.030534 | 2.696086 | 0.830063 |
| 4 | 6 | 0 | -2.990033 | 0.219505 | 0.263221 |
| 5 | 6 | 0 | -1.399833 | 1.577636 | -2.143396 |
| 6 | 6 | 0 | -1.299228 | -2.190681 | 1.500674 |
| 7 | 6 | 0 | -2.137945 | -1.588954 | -1.546673 |
| 8 | 6 | 0 | 0.848604 | -0.793381 | -2.600843 |
| 9 | 6 | 0 | 0.905974 | -2.782059 | -0.756894 |
| 10 | 6 | 0 | 1.211036 | -1.497330 | 2.170208 |
| 11 | 6 | 0 | 2.959906 | -0.181291 | -0.188127 |
| 12 | 6 | 0 | -0.991901 | 0.765804 | 2.686668 |
| 13 | 14 | 0 | 0.208681 | 1.974767 | 1.987815 |
| 14 | 14 | 0 | 2.251321 | 1.553465 | -0.237314 |
| 15 | 14 | 0 | -0.565847 | 2.667342 | -0.883617 |
| 16 | 14 | 0 | -2.424129 | 0.262759 | -1.482876 |
| 17 | 14 | 0 | -2.412908 | -1.530344 | 0.279064 |
| 18 | 14 | 0 | -2.142227 | 1.326037 | 1.362066 |
| 19 | 14 | 0 | -0.495421 | -0.931127 | 2.582225 |
| 20 | 14 | 0 | 0.406703 | -2.622124 | 0.951062 |
| 21 | 14 | 0 | 2.288361 | -0.232149 | 1.532076 |
| 22 | 14 | 0 | -0.417850 | -2.017198 | -1.830280 |
| 23 | 14 | 0 | 2.022164 | -1.345698 | -1.181315 |
| 24 | 14 | 0 | 0.315537 | 1.005954 | -2.468647 |
| 25 | 8 | 0 | 0.317507 | -0.310844 | -1.112955 |
| 26 | 3 | 0 | 5.416052 | 0.155594 | -0.510389 |

**Table S12. Cartesian Coordinates of Li@Se-Ge_12_C_12_ Endohedral Doped** **Nanocage.**

| **Center**  **Number** | **Atomic**  **Number** | **Atomic**  **Type** | **Coordinates (Angstroms)** | | |
| --- | --- | --- | --- | --- | --- |
|  |  |  | **X** | **Y** | **Z** |
| 1 | 32 | 0 | 0.567062 | 0.966338 | -2.643773 |
| 2 | 6 | 0 | 1.315640 | -0.823136 | -2.685259 |
| 3 | 6 | 0 | 1.146922 | 2.410386 | -1.604256 |
| 4 | 6 | 0 | 3.182078 | 0.162352 | 0.063119 |
| 5 | 6 | 0 | 1.182165 | -0.329024 | 2.847598 |
| 6 | 6 | 0 | 1.078100 | 2.661874 | 1.209147 |
| 7 | 6 | 0 | -1.221747 | -2.408383 | 1.603265 |
| 8 | 6 | 0 | -1.384012 | 0.822446 | 2.687307 |
| 9 | 6 | 0 | -2.002666 | 2.430230 | -0.263775 |
| 10 | 6 | 0 | -3.150601 | -0.138453 | -0.062426 |
| 11 | 6 | 0 | -1.154599 | -2.658919 | -1.206106 |
| 12 | 6 | 0 | -1.249383 | 0.328922 | -2.848945 |
| 13 | 6 | 0 | 1.923317 | -2.403741 | 0.259355 |
| 14 | 32 | 0 | -0.187321 | 2.875863 | -0.259512 |
| 15 | 32 | 0 | -2.358833 | 0.941790 | -1.466490 |
| 16 | 32 | 0 | -2.418602 | 1.174465 | 1.163277 |
| 17 | 32 | 0 | 0.442452 | 1.422620 | 2.459032 |
| 18 | 32 | 0 | -0.649822 | -0.973269 | 2.656654 |
| 19 | 32 | 0 | -2.308436 | -1.800204 | 0.106382 |
| 20 | 32 | 0 | 0.092727 | -2.876241 | 0.259352 |
| 21 | 32 | 0 | -0.525394 | -1.429563 | -2.466457 |
| 22 | 32 | 0 | 2.242250 | 1.848376 | -0.108822 |
| 23 | 32 | 0 | 2.291656 | -0.953223 | 1.483010 |
| 24 | 32 | 0 | 2.371696 | -1.200456 | -1.190908 |
| 25 | 3 | 0 | 5.113912 | 0.045139 | 0.082650 |
| 26 | 34 | 0 | 0.022502 | -0.010313 | 0.000645 |

**Table S13. Cartesian Coordinates of Li@Se-Si_12_C_12_ Endohedral Doped** **Nanocage.**

| **Center**  **Number** | **Atomic**  **Number** | **Atomic**  **Type** | **Coordinates (Angstroms)** | | |
| --- | --- | --- | --- | --- | --- |
|  |  |  | **X** | **Y** | **Z** |
| 1 | 6 | 0 | -0.369024 | -0.300428 | -2.488944 |
| 2 | 6 | 0 | -1.053471 | -2.718219 | -0.653304 |
| 3 | 6 | 0 | -2.849150 | 0.032645 | -0.608308 |
| 4 | 6 | 0 | -1.655203 | 1.618857 | 1.871765 |
| 5 | 6 | 0 | -1.669734 | -1.747959 | 1.756374 |
| 6 | 6 | 0 | 1.257057 | 2.739075 | 0.686383 |
| 7 | 6 | 0 | 0.577135 | 0.332058 | 2.545609 |
| 8 | 6 | 0 | 1.637293 | -2.292380 | 1.228257 |
| 9 | 6 | 0 | 3.045904 | -0.003437 | 0.639306 |
| 10 | 6 | 0 | 1.882705 | 1.782591 | -1.727202 |
| 11 | 6 | 0 | 1.865880 | -1.596295 | -1.842781 |
| 12 | 6 | 0 | -1.438799 | 2.324834 | -1.197277 |
| 13 | 14 | 0 | -1.794494 | 0.692728 | -1.972288 |
| 14 | 14 | 0 | 0.032345 | -2.029237 | -1.906418 |
| 15 | 14 | 0 | -2.422752 | -1.622153 | 0.055472 |
| 16 | 14 | 0 | -1.225252 | -0.129084 | 2.397672 |
| 17 | 14 | 0 | 0.177593 | 2.049803 | 1.943226 |
| 18 | 14 | 0 | -2.434286 | 1.669199 | 0.254511 |
| 19 | 14 | 0 | 0.374098 | 2.607756 | -0.945724 |
| 20 | 14 | 0 | 2.632346 | 1.654544 | -0.024432 |
| 21 | 14 | 0 | 1.440681 | 0.157799 | -2.359835 |
| 22 | 14 | 0 | 1.988075 | -0.658243 | 2.003308 |
| 23 | 14 | 0 | 2.638600 | -1.642388 | -0.220539 |
| 24 | 14 | 0 | -0.170354 | -2.585099 | 0.979966 |
| 25 | 34 | 0 | -0.260097 | -0.058205 | -0.042514 |
| 26 | 3 | 0 | -5.284214 | -0.455942 | -0.894217 |

**Table 14. Cartesian Coordinates of Pristine Li^+^@Ge_12_C_12_** **Nanocage.**

| **Center**  **Number** | **Atomic**  **Number** | **Atomic**  **Type** | **Coordinates (Angstroms)** | | |
| --- | --- | --- | --- | --- | --- |
|  |  |  | **X** | **Y** | **Z** |
| 1 | 32 | 0 | 0.568824 | 0.965519 | -2.643725 |
| 2 | 6 | 0 | 1.317226 | -0.824029 | -2.685211 |
| 3 | 6 | 0 | 1.148825 | 2.409510 | -1.604208 |
| 4 | 6 | 0 | 3.183761 | 0.161276 | 0.063167 |
| 5 | 6 | 0 | 1.183800 | -0.329904 | 2.847646 |
| 6 | 6 | 0 | 1.080027 | 2.661004 | 1.209195 |
| 7 | 6 | 0 | -1.220316 | -2.409028 | 1.603313 |
| 8 | 6 | 0 | -1.382264 | 0.821817 | 2.687355 |
| 9 | 6 | 0 | -2.000761 | 2.429662 | -0.263727 |
| 10 | 6 | 0 | -3.148948 | -0.138908 | -0.062378 |
| 11 | 6 | 0 | -1.153192 | -2.659570 | -1.206058 |
| 12 | 6 | 0 | -1.247684 | 0.328280 | -2.848897 |
| 13 | 6 | 0 | 1.924749 | -2.404694 | 0.259403 |
| 14 | 32 | 0 | -0.185373 | 2.875117 | -0.259464 |
| 15 | 32 | 0 | -2.357074 | 0.941257 | -1.466442 |
| 16 | 32 | 0 | -2.416820 | 1.173938 | 1.163325 |
| 17 | 32 | 0 | 0.444258 | 1.421813 | 2.459080 |
| 18 | 32 | 0 | -0.648251 | -0.973969 | 2.656702 |
| 19 | 32 | 0 | -2.306946 | -1.800742 | 0.106430 |
| 20 | 32 | 0 | 0.094112 | -2.877014 | 0.259400 |
| 21 | 32 | 0 | -0.523867 | -1.430276 | -2.466409 |
| 22 | 32 | 0 | 2.244097 | 1.847392 | -0.108774 |
| 23 | 32 | 0 | 2.293229 | -0.954211 | 1.483058 |
| 24 | 32 | 0 | 2.373245 | -1.201452 | -1.190860 |
| 25 | 3 | 0 | 5.115583 | 0.043875 | 0.082698 |

**Table S15. Cartesian Coordinates of Pristine Li^+^@Si_12_C_12_** **Nanocage.**

| **Center**  **Number** | **Atomic**  **Number** | **Atomic**  **Type** | **Coordinates (Angstroms)** | | |
| --- | --- | --- | --- | --- | --- |
|  |  |  | **X** | **Y** | **Z** |
| 1 | 6 | 0 | -2.034758 | -1.580251 | -1.543815 |
| 2 | 6 | 0 | 0.202775 | -2.907119 | 0.321040 |
| 3 | 6 | 0 | -2.188122 | -1.238834 | 1.637509 |
| 4 | 6 | 0 | -0.660048 | 1.436946 | 2.498552 |
| 5 | 6 | 0 | 0.921810 | -1.343027 | 2.409162 |
| 6 | 6 | 0 | -0.360042 | 2.914814 | -0.329067 |
| 7 | 6 | 0 | 1.859963 | 1.558565 | 1.504052 |
| 8 | 6 | 0 | 2.751343 | -1.006629 | -0.217527 |
| 9 | 6 | 0 | 2.043165 | 1.233572 | -1.607188 |
| 10 | 6 | 0 | -1.058570 | 1.344379 | -2.418600 |
| 11 | 6 | 0 | 0.509034 | -1.428845 | -2.476648 |
| 12 | 6 | 0 | -2.841771 | 0.967815 | 0.209346 |
| 13 | 14 | 0 | -2.649359 | -0.817428 | -0.074002 |
| 14 | 14 | 0 | -0.425317 | -2.371154 | -1.236497 |
| 15 | 14 | 0 | -0.610511 | -2.023027 | 1.692677 |
| 16 | 14 | 0 | 0.858090 | 0.424052 | 2.472412 |
| 17 | 14 | 0 | 0.268826 | 2.360632 | 1.224748 |
| 18 | 14 | 0 | -2.016998 | 0.572816 | 1.786740 |
| 19 | 14 | 0 | -1.782531 | 1.908272 | -0.846413 |
| 20 | 14 | 0 | 0.471122 | 2.016639 | -1.677015 |
| 21 | 14 | 0 | -1.003771 | -0.413077 | -2.493005 |
| 22 | 14 | 0 | 2.788628 | 0.946350 | 0.082154 |
| 23 | 14 | 0 | 1.837870 | -0.537361 | -1.728119 |
| 24 | 14 | 0 | 1.622715 | -1.889195 | 0.828729 |
| 25 | 3 | 0 | 4.702876 | -0.731190 | -0.124877 |

**Table S16. Cartesian Coordinates of Li^+^@O-Ge_12_C_12_ Endohedral Doped** **Nanocage.**

| **Center**  **Number** | **Atomic**  **Number** | **Atomic**  **Type** | **Coordinates (Angstroms)** | | |
| --- | --- | --- | --- | --- | --- |
|  |  |  | **X** | **Y** | **Z** |
| 1 | 32 | 0 | 2.548227 | -1.184514 | -0.919022 |
| 2 | 6 | 0 | 3.115270 | 0.251222 | 0.239533 |
| 3 | 6 | 0 | 1.551876 | -0.982914 | -2.471233 |
| 4 | 6 | 0 | 1.290038 | 2.282519 | -1.739201 |
| 5 | 6 | 0 | -1.866952 | 2.054095 | -0.534003 |
| 6 | 6 | 0 | -1.019538 | -0.039239 | -3.020882 |
| 7 | 6 | 0 | -1.539104 | 0.942502 | 2.618975 |
| 8 | 6 | 0 | -3.155170 | -0.393992 | -0.226208 |
| 9 | 6 | 0 | -0.885063 | -2.773840 | -1.023515 |
| 10 | 6 | 0 | -1.136995 | -2.297341 | 1.711643 |
| 11 | 6 | 0 | 1.058600 | -0.050938 | 2.884154 |
| 12 | 6 | 0 | 2.035832 | -2.286937 | 0.581785 |
| 13 | 6 | 0 | 1.031133 | 2.769219 | 1.064446 |
| 14 | 32 | 0 | -0.235262 | -1.700311 | -2.399119 |
| 15 | 32 | 0 | 0.274110 | -2.865683 | 0.520147 |
| 16 | 32 | 0 | -2.201962 | -1.966258 | 0.135243 |
| 17 | 32 | 0 | -2.266799 | 0.505252 | -1.722437 |
| 18 | 32 | 0 | -2.518554 | 0.981836 | 1.013774 |
| 19 | 32 | 0 | -0.708749 | -0.805570 | 2.740280 |
| 20 | 32 | 0 | 0.288665 | 1.632058 | 2.359827 |
| 21 | 32 | 0 | 2.308090 | -0.730123 | 1.692348 |
| 22 | 32 | 0 | 0.753529 | 0.753583 | -2.686810 |
| 23 | 32 | 0 | -0.086251 | 2.850964 | -0.508757 |
| 24 | 32 | 0 | 2.304642 | 1.881688 | -0.119543 |
| 25 | 8 | 0 | -1.042592 | 1.393677 | -0.257418 |
| 26 | 3 | 0 | -3.082900 | 4.236982 | -0.614475 |

**Table S17. Cartesian Coordinates of Li^+^@O-Si_12_C_12_ Endohedral Doped** **Nanocage.**

| **Center**  **Number** | **Atomic**  **Number** | **Atomic**  **Type** | **Coordinates (Angstroms)** | | |
| --- | --- | --- | --- | --- | --- |
|  |  |  | **X** | **Y** | **Z** |
| 1 | 6 | 0 | 1.881881 | 1.564869 | 1.560931 |
| 2 | 6 | 0 | 1.163805 | 2.285859 | -1.460635 |
| 3 | 6 | 0 | -1.030534 | 2.696086 | 0.830063 |
| 4 | 6 | 0 | -2.990033 | 0.219505 | 0.263221 |
| 5 | 6 | 0 | -1.399833 | 1.577636 | -2.143396 |
| 6 | 6 | 0 | -1.299228 | -2.190681 | 1.500674 |
| 7 | 6 | 0 | -2.137945 | -1.588954 | -1.546673 |
| 8 | 6 | 0 | 0.848604 | -0.793381 | -2.600843 |
| 9 | 6 | 0 | 0.905974 | -2.782059 | -0.756894 |
| 10 | 6 | 0 | 1.211036 | -1.497330 | 2.170208 |
| 11 | 6 | 0 | 2.959906 | -0.181291 | -0.188127 |
| 12 | 6 | 0 | -0.991901 | 0.765804 | 2.686668 |
| 13 | 14 | 0 | 0.208681 | 1.974767 | 1.987815 |
| 14 | 14 | 0 | 2.251321 | 1.553465 | -0.237314 |
| 15 | 14 | 0 | -0.565847 | 2.667342 | -0.883617 |
| 16 | 14 | 0 | -2.424129 | 0.262759 | -1.482876 |
| 17 | 14 | 0 | -2.412908 | -1.530344 | 0.279064 |
| 18 | 14 | 0 | -2.142227 | 1.326037 | 1.362066 |
| 19 | 14 | 0 | -0.495421 | -0.931127 | 2.582225 |
| 20 | 14 | 0 | 0.406703 | -2.622124 | 0.951062 |
| 21 | 14 | 0 | 2.288361 | -0.232149 | 1.532076 |
| 22 | 14 | 0 | -0.417850 | -2.017198 | -1.830280 |
| 23 | 14 | 0 | 2.022164 | -1.345698 | -1.181315 |
| 24 | 14 | 0 | 0.315537 | 1.005954 | -2.468647 |
| 25 | 8 | 0 | 0.317507 | -0.310844 | -1.112955 |
| 26 | 3 | 0 | 5.416052 | 0.155594 | -0.510389 |

**Table S18. Cartesian Coordinates of Li^+^@Se-Ge_12_C_12_ Endohedral Doped Nanocage.**

| **Center**  **Number** | **Atomic**  **Number** | **Atomic**  **Type** | **Coordinates (Angstroms)** | | |
| --- | --- | --- | --- | --- | --- |
|  |  |  | **X** | **Y** | **Z** |
| 1 | 32 | 0 | 0.715611 | -1.873859 | 2.096650 |
| 2 | 6 | 0 | 2.351522 | -0.795648 | 1.901107 |
| 3 | 6 | 0 | -0.052376 | -3.063129 | 0.849998 |
| 4 | 6 | 0 | 2.422244 | -1.725638 | -1.428219 |
| 5 | 6 | 0 | 0.119477 | 0.485850 | -3.033250 |
| 6 | 6 | 0 | -1.200804 | -2.541187 | -1.744114 |
| 7 | 6 | 0 | 0.008155 | 3.115450 | -0.809983 |
| 8 | 6 | 0 | -2.512828 | 0.862780 | -1.936179 |
| 9 | 6 | 0 | -2.811673 | -1.059874 | 0.907575 |
| 10 | 6 | 0 | -2.238016 | 1.670238 | 1.368251 |
| 11 | 6 | 0 | 1.095407 | 2.411248 | 1.709154 |
| 12 | 6 | 0 | -0.196345 | -0.456647 | 3.050254 |
| 13 | 6 | 0 | 2.932461 | 1.135905 | -0.910175 |
| 14 | 32 | 0 | -1.781036 | -2.450121 | 0.141859 |
| 15 | 32 | 0 | -1.820631 | -0.021608 | 2.206903 |
| 16 | 32 | 0 | -2.934951 | 0.609854 | -0.106891 |
| 17 | 32 | 0 | -1.361826 | -0.701882 | -2.335278 |
| 18 | 32 | 0 | -0.835586 | 1.900025 | -1.999130 |
| 19 | 32 | 0 | -0.683814 | 2.645503 | 0.955268 |
| 20 | 32 | 0 | 1.723786 | 2.407087 | -0.124817 |
| 21 | 32 | 0 | 1.248323 | 0.704444 | 2.476976 |
| 22 | 32 | 0 | 0.628092 | -2.457326 | -0.881158 |
| 23 | 32 | 0 | 1.774529 | 0.080593 | -2.099774 |
| 24 | 32 | 0 | 2.931080 | -0.528838 | 0.139590 |
| 25 | 3 | 0 | 3.761892 | -2.771717 | -2.373447 |
| 26 | 34 | 0 | 0.055779 | -0.057791 | -0.219780 |

**Table S19. Cartesian Coordinates of Li^+^@Se-Si_12_C_12_ Endohedral Doped Nanocage.**

| **Center**  **Number** | **Atomic**  **Number** | **Atomic**  **Type** | **Coordinates (Angstroms)** | | |
| --- | --- | --- | --- | --- | --- |
|  |  |  | **X** | **Y** | **Z** |
| 1 | 6 | 0 | -0.369024 | -0.300428 | -2.488944 |
| 2 | 6 | 0 | -1.053471 | -2.718219 | -0.653304 |
| 3 | 6 | 0 | -2.849150 | 0.032645 | -0.608308 |
| 4 | 6 | 0 | -1.655203 | 1.618857 | 1.871765 |
| 5 | 6 | 0 | -1.669734 | -1.747959 | 1.756374 |
| 6 | 6 | 0 | 1.257057 | 2.739075 | 0.686383 |
| 7 | 6 | 0 | 0.577135 | 0.332058 | 2.545609 |
| 8 | 6 | 0 | 1.637293 | -2.292380 | 1.228257 |
| 9 | 6 | 0 | 3.045904 | -0.003437 | 0.639306 |
| 10 | 6 | 0 | 1.882705 | 1.782591 | -1.727202 |
| 11 | 6 | 0 | 1.865880 | -1.596295 | -1.842781 |
| 12 | 6 | 0 | -1.438799 | 2.324834 | -1.197277 |
| 13 | 14 | 0 | -1.794494 | 0.692728 | -1.972288 |
| 14 | 14 | 0 | 0.032345 | -2.029237 | -1.906418 |
| 15 | 14 | 0 | -2.422752 | -1.622153 | 0.055472 |
| 16 | 14 | 0 | -1.225252 | -0.129084 | 2.397672 |
| 17 | 14 | 0 | 0.177593 | 2.049803 | 1.943226 |
| 18 | 14 | 0 | -2.434286 | 1.669199 | 0.254511 |
| 19 | 14 | 0 | 0.374098 | 2.607756 | -0.945724 |
| 20 | 14 | 0 | 2.632346 | 1.654544 | -0.024432 |
| 21 | 14 | 0 | 1.440681 | 0.157799 | -2.359835 |
| 22 | 14 | 0 | 1.988075 | -0.658243 | 2.003308 |
| 23 | 14 | 0 | 2.638600 | -1.642388 | -0.220539 |
| 24 | 14 | 0 | -0.170354 | -2.585099 | 0.979966 |
| 25 | 34 | 0 | -0.260097 | -0.058205 | -0.042514 |
| 26 | 3 | 0 | -5.284214 | -0.455942 | -0.894217 |

**Table S20.** **ML-Predicted V­_cell_ Vs DFT-Predicted V_cell­_ of the Studied Nanocages.**

| **DFT-Predicted V_cell_ (V)** | **ML-Predicted V_cell_ (V)** | | | |
| --- | --- | --- | --- | --- |
| Y_train | Linear | Lasso | Ridge | ElasticNet |
| 1.49 | 1.4796 | 1.4890 | 1.4626 | 1.4684 |
| 0.06 | 0.0581 | 0.0600 | 0.0567 | 0.0584 |
| 0.14 | 0.1404 | 0.1399 | 0.1410 | 0.1406 |
| -0.27 | -0.2701 | -0.2699 | -0.2691 | -0.2688 |

**Table S20** shows that our ML-predicted V_cells_ are closer to the DFT-predicted V_cells_ (Y_train). Lasso Regression exhibited the best accuracy due to its L1 regularization technique, which penalizes cost function by setting coefficients of unimportant features to zero, while selecting important features.
